# Comparison of synthetic and post-synthetic molecular genetic methods of reducing xylan glucuronidation in hybrid aspen for biorefinery applications

**DOI:** 10.1186/s13068-026-02830-x

**Published:** 2026-10-15

**Authors:** Pramod Sivan, János Urbancsok, Madhavi L. Gandla, Evgeniy N. Donev, Marta Derba-Maceluch, Melissa Roach, Leif J. Jönsson, Francisco Vilaplana, Ewa J. Mellerowicz

**Affiliations:** 1 Department of Forest Genetics and Plant Physiology, Umeå Plant Science Centre, Swedish University of Agricultural Sciences, 901 83 Umeå, Sweden; 2 Department of Chemistry, Umeå University, 901 87 Umeå, Sweden; 3 Division of Glycoscience, Department of Chemistry, KTH Royal Institute of Technology, AlbaNova University Centre, 106 91 Stockholm, Sweden; 4 Wallenberg Wood Science Centre (WWSC), KTH Royal Institute of Technology, 100 44 Stockholm, Sweden; 5 Present Address: HUN-REN Biological Research Centre, Institute of Plant Biology, 6726 Szeged, Hungary; 6 Present Address: Faculty of Science - Biological Sciences, University of Alberta, Edmonton, Canada

**Keywords:** Secondary cell wall, Glucuronoxylan, Glucuronidation, GUX1, α-glucuronidase, Xylan acetylation, Wood chemistry, Saccharification, Transgenic trees, *Populus*

## Abstract

**Background:**

The pattern of xylan backbone substitution by 4-*O*-methylglucuronic acid (mGlcA) and *O*-acetyl groups generated during biosynthesis in the Golgi apparatus plays a key role in molecular interactions between secondary cell wall polymers in hardwoods, thus regulating biorefinery properties of woody biomass. This study aims at comparing the effects of two different approaches to reduce xylan glucuronidation, targeting either xylan biosynthesis by RNAi suppression of *GLUCURONIC SUBSTITUTION OF XYLAN1* genes (denoted Gux1RNAi) or by xylan post-synthetic modification in the secondary cell wall by apoplastic wood-specific expression of a fungal α-glucuronidase from family GH67 (denoted WP:GH67) in hybrid aspen (Populus tremula L. x tremuloides Michx.). The effects of reducing xylan glucuronidation by these two approaches on xylan acetylation and solubility, and recalcitrance of wood to enzymatic hydrolysis were compared.

**Results:**

Both approaches reduced mGlcA/xylose (Xyl) ratio by approx. 30% without altering Xyl content in alkali extracts, while xylan acetyl content in subcritical water extracts was increased only in Gux1RNAi lines. The Gux1RNAi lines showed reduced glucuronidation in the major xylan domain containing evenly-spaced mGlcA, whereas the WP:GH67 lines were affected in both odd and even glucuronidation patterns. Interestingly, the arrays containing mGlcA every second xylosyl unit were not affected by any modification, whereas an increase in xylo-oligosaccharides containing *O*-2,3 diacetylated Xyl was found in Gux1RNAi lines only. Neither modification consistently affected glucose or Xyl yields in saccharification without or with acid pretreatment, whereas xylan extractability in subcritical water was increased in Gux1RNAi, but not in WP:GH67 lines.

**Conclusion:**

Our study supports the hypothesis that acetylation competes with glucuronidation by GUX1 during biosynthesis, and increased acetylation caused by *GUX1* suppression rather than reduced xylan glucuronidation per se improves xylan extractability. The 30% reduction in glucuronidation did not affect enzymatic saccharification, supporting the concept that cell wall structure and recalcitrance in transgenic lines are maintained by xylan domains which were not affected by either transgenic modification.

**Supplementary Information:**

The online version contains supplementary material available at https://doi.org/10.1186/s13068-026-02830-x.

## Highlights

Reducing xylan glucuronidation either by targeting xylan glucuronosyltransferases in the Golgi or by post-synthetic hydrolysis of glucuronic acid side chains in secondary cell walls has different effects on xylan acetylation and consequently on xylan extractability.

**Supplementary Information:**

The online version contains supplementary material available at https://doi.org/10.1186/s13068-026-02830-x.

## Background

Plant cell walls are deposited in discrete layers including the middle lamella, primary wall (PW) and additional layers found in some cell types like wood cells, namely the secondary wall (SW) layers S1, S2 and S3, and the gelatinous layer (G-layer) found in tension wood fibers, which together constitute the bulk of wood biomass. These different cell wall layers contain specific proportions of cellulose, pectins, different types of hemicelluloses (xylan, mannan, xyloglucan and, in some species, also the mixed linked *β*-glycan) and lignin [1]. Xylan is the major hemicellulose in SW layers in dicotyledons, both in herbaceous plants and hardwood species, where it has a form of acetylated glucuronoxylan (GX). Structurally, GX is composed of *β*-(1,4)-linked D-xylopyranose units substituted with 4-*O*-methylglucuronic acid (mGlcA) at the *O*−2 position and/or *O*-acetyl groups at positions *O*−2, and/or *O*−3 of xylan backbone [2–4]. The spacing pattern of mGlcA and acetyl substitutions determines molecular interactions between SW polymers, thereby influencing the physical and chemical properties of hardwood biomass [5]. Hardwood GX has two different domains with distinct substitution patterns: a major domain with even mGlcA and *O*-acetyl spacing on the xylosyl units that allows a two-fold screw conformation when interacting with cellulose microfibrils (CMFs) and a minor domain characterized by a denser and uneven glucuronidation spacing and reduced acetylation [6–10]. The minor domain is believed to take a three-fold screw conformation in the space between the CMFs [11], and can be highly acetylated affecting benzyl ether linkages with lignin [12] and promoting interactions with hydrophobic pockets in the space between CMFs [3]. Therefore, GX is proposed to play a key role in tailoring the molecular architecture of SW of hardwoods.

The biosynthesis of GX takes place in the Golgi. GX backbone is elongated in Golgi lumen by a xylan synthase protein complex that has different composition during PW and SW xylan biosynthesis [13]. The PW xylan synthase complex contains xylan synthase IRX10L from the glycosyl transferase (GT) 47 family that has xylan UDP-Xyl transferase activity and two accessory proteins, IRX9L and IRX14 from the GT43 family, which are needed for the xylan synthase activity [14–16]. The SW xylan synthase complex contains the isoforms IRX10 (instead of IRX10L), IRX9 (instead of IRX9L) and IRX14 (similar to the PW complex) [17–20]. The reducing end of xylan backbone in hardwoods is terminated by a tetrasaccharide [xylose (Xyl)-rhamnose (Rha)-galacturonic acid (GalA)-Xyl] sequence made by FRA8/IRX7 from the GT47 family [21, 22], two GT8 members: IRX8/GAUT12 [22] and PARVUS/GATL1 [23, 24], and the GT106 member FRA9 [25]. The glucuronic acid (GlcA) is added to the xylan backbone by GLUCURONIC ACID SUBSTITUTION OF XYLAN (GUX) proteins (GUX1-3) from family GT8 [26, 27]. GUX1 and GUX3 are involved in glucuronidation of the major domain generating an even spacing, while GUX2 is responsible for GlcA substitution of minor domain with no preference for odd or even spacing [6]. Both GUX1 and GUX2 are responsible for SW xylan glucuronidation, whereas GUX3 has a role in xylan glucuronidation in PW [15]. A substantial part of GlcA side chains in PW xylan carries an arabinopyranosyl substitution at *O*−2, which is added by the xylan 2-*O* arabinopyranosyltransferase 1 (XAPT1) from the *MUR3* clade of GT47 [28, 29]. Methylation of GlcA at *O*−4 is accomplished by GLUCURONOXYLAN METHYL TRANSFERASE 1 (GXM1), a DUF579 family protein and it is more prominent in SW than in PW [30, 31]. Addition of acetyl groups requires at least two protein families: REDUCED WALL ACETYLATION (RWA1-RWA4 in *Arabidopsis*) facilitating transfer of acetyl-CoA to Golgi [32–34], and TRICHOME BIREFRINGENCE-LIKE (TBL) family members, such as TBL29/ESKIMO1 [11, 35], TBL3, TBL31 [36], TBL32, TBL33 [37], TBL34 and TBL35 [38], involved in transfer of *O*-acetyl groups into xylan backbone at different positions.

Following biosynthesis, the GX acetyl substitutions have been shown to be modified either in the Golgi or after cell wall deposition. Certain GDSL-motif esterase/acyltransferase/lipase proteins (GELPs) acting still in Golgi or *in muro,* but attached to plasma membrane have been shown to deacetylate xylan [39–41]. After secretion to the cell wall, the GX backbone can be *endo*-hydrolyzed or modified via transglycosylation by GH10 family proteins [42, 43] or *exo-*hydrolyzed by *β*-xylosidases from family GH3 [44]. These GX post-synthetic modifications affect cell wall assembly, integrity and stress signaling.

Several biotechnological attempts have been reported to modify xylan structure to enhance the efficacy of biochemical conversion of wood biomass. Post-synthetic modifications of GX by overexpression of microbial xylan-active enzymes *in muro* have been demonstrated to have potential to reduce recalcitrance of lignocellulosic biomass, but frequently these modifications affect also stress signaling, plant growth and development [45–50]. Targeting native biosynthetic enzymes have been extensively used for the same purpose with many promising results, but often different off-target effects were also observed [51–53]. Therefore, the objective assessment of different biotechnological procedures is required for achieving optimal results.

Reducing GX glucuronidation or influencing its pattern have been two main biotechnological goals of xylan modification for improved biomass properties. Decreased xylan glucuronidation in *gux1gux2* or *gux1gux2gux3* mutants in *Arabidopsis* [27, 54] and in silenced *ScGUX2* lines in sugarcane [55] improved sugar release during enzymatic saccharification of biomass suggesting the presence of glucuronidation plays an important role in biomass recalcitrance. In contrast, our previous analyses in aspen with reduced GX glucuronidation (by 20–30%) caused by ectopic expression of an *Aspergillus niger* GH67 α-glucuronidase (35S:GH67), did not reveal any change in enzymatic sugar yields from aspen wood [56]. Since the acetylation and glucuronidation processes of xylan could be dynamically regulated during biosynthesis in the Golgi via competing activities between xylan acetyl transferase and xylan UDP-GlcA transferases, it is possible that changes in acetylation in the *gux* mutants [35, 57] affect recalcitrance of their biomass. Moreover, a substantial increase in Xyl in matrix polysaccharides of *gux1gux2* double mutants indicating increased xylan content [58] was another off-target effect that could have contributed to higher Xyl yields in enzymatic saccharification of these mutants [54]. Thus, the reduction in glucuronidation might have different effects on biomass recalcitrance when using synthetic and post-synthetic genetic strategies due to their different associated secondary effects. Therefore, in this study we compared these two molecular genetic methods for reducing glucuronidation in aspen to improve wood biorefinery traits. Glucuronidation was reduced by: i) downregulating native *Populus GUX1* genes (Gux1RNAi) and ii) expressing *Aspergillus niger* GH67 α-glucuronidase (WP:GH67) specifically in the wood using the *P. trichocarpa GT43B* promoter [16] called “wood-specific promoter” (WP). While the first strategy targeted the process of GX biosynthesis in the Golgi, the latter one targeted GX in secondary cell walls where mGlcA side chains were removed from GX by the apoplastic GH67*.* To evaluate these two strategies, we compared their effects on GX structure, xylan extractability and wood saccharification.

## Materials and methods

### Plant material

Transgenic hybrid aspen (Populus tremula L. x tremuloides Michx.), clone T89, lines expressing *Aspergillus niger* GH67 α-glucuronidase (35S:GH67 and WP:GH67) were described in previous publications [50, 56]. For the suppression of glucuronidation at synthesis stage, a 207 nt cDNA sequence (GGAACATATTAACGAGATCGAATCCTATAATGGTGGAGATCAAGGCTATCTGAATGAGATCTTTACATGGTGGCATCGTATTCCAAAACACATGAACTTCTTAAAACATTTCTGGATTGGTGATGAGGAAGAGGTTAAGCAAAAGAAAACTAGCCTTTTCGGAGCCGAGCCTCCAATTCTCTATGCCCTTCACTATCTTGGTGTAAA) cloned from hybrid aspen using the primers CACCGGAACATATTAACGAGATCGA (forward) and TTTACACCAAGATAGTGAAGGACATA (reverse) listed in Supplementary Table S1 was used to silence both *PtGUX1A* (*Potri.007G107200/Potra2n7c15752*) and *PtGUX1B* (*Potri.005G061600/Potra2n5c12319*) genes. The sequence corresponds to regions in the last exon of *PtGUX1A* and *PtGUX1B* genes with 93% sequence identity between them in *P. tremula* (sequences from plantgenie.org). The amplified 207 bp fragment was cloned into the binary RNAi vector pK7GWIWG2(I) [59] with 35S promoter and introduced into competent *Agrobacterium tumefaciens* (Smith and Townsend) Conn strain GV3101 by electroporation. Twenty transgenic lines carrying each construct were screened in vitro by semi-quantitative reverse transcription polymerase chain reaction (RT-PCR) and five–six were selected for the greenhouse experiment. Out of these lines, the two of each set that expressed/suppressed the target gene in the wood-forming tissues most strongly were selected for phenotyping and more detailed experiments.

### Plant growth in the greenhouse and tissue collection

Six in vitro propagated saplings per each line along with 24 wild-type trees were planted in soil (K-jord, Hasselfors Garden AB, Örebro, Sweden) in 7 L plastic pots, watered to 25–30% (v:v) soil moisture content, covered with transparent 8 L plastic bags, and grown for nine weeks in the phenotyping platform (WIWAM Conveyor, custom designed by SMO, Eeklo, Belgium) as described by Wang et al. [60] under 18 h/6 h (day/night) light regime with 160–230 µmol m^−2^ s ^−1^ light intensity during the day, 22 °C/18 °C temperature, and the average air relative humidity of 60%. White light (FL300 LED Sunlight v1.1) and far-red light (FL100 LED custom-made, 725–735 nm) lamps from Senmatic A/S (Søndersø, Denmark) were used for illumination. After two weeks the bags were removed, and plants were watered automatically based on weight and their height was automatically recorded.

At harvest, the trees were photographed, and their stem diameters (at the base) and aboveground fresh weights were recorded. For RNA extraction, a 30 cm-long stem segment above internode 37 was dissected, debarked, frozen in liquid nitrogen and stored at − 70 °C. Frozen wood core was scraped with a scalpel into a pre-cooled mortar and ground in liquid nitrogen to obtain frozen developing xylem powder.

### RNA analyses

To isolate total RNA, frozen cambium/phloem and xylem powders (approximately 100 mg) were extracted with CTAB/chloroform:isoamyl alcohol (24:1), followed by precipitation with LiCl and sodium acetate/ethanol [61]. Three trees of each genotype were analyzed by RT-qPCR procedures as described previously [47]. *UBQ-L* (*Potri.005G198700*) and *ACT11* (*Potri.006G192700*) were selected as reference genes. The primer sequences are listed in Supplementary Table S1. The relative expression level was calculated according to Pfaffl [62].

For transcriptomics, RNA from five trees per transgenic line and ten trees from wild type was purified as described previously [63] and used for cDNA preparation and sequencing at Novogene Co., Ltd. (Cambridge, United Kingdom). Quality control and mapping to the *P. tremula* transcriptome (v.2.2), retrieved from the PlantGenIE [64] (https://plantgenie.org and to sequences used in fungal vectors were carried out by Novogene. Raw counts were used for differential expression analysis in R (v.3.4.0) using Bioconductor (v.3.4) DESeq2 package (v.1.16.1), as previously described [65]. The best BLAST hits were identified as *Arabidopsis thaliana* (v.11.0).

### Wood sample preparation for cell wall chemical analysis

For the pyrolysis, the wood powder was obtained by filing the freeze-dried wood from three trees per line and sieving the sawdust with Retsch AS 200 analytical sieve shaker (Retsch GmbH, Haan, Germany) to the particle size between 50 and 100 µm. For the remaining cell wall analyses, the wood from six trees per line was combined. The pith was removed from debarked and freeze-dried stem segments and the segments were ground using Retsch Ultra Centrifugal Mill ZM 200 (Retsch GmbH, Haan, Germany) equipped with a 0.5 mm ring sieve. The resulting wood powder was then sieved by Retsch AS 200 vibratory sieve shaker to isolate powder with particle sizes of 50–100 μm and 100–500 μm.

### Hemicellulose extraction

Hemicelluloses were extracted using two different methods; to extract acetylated xylan, subcritical water extraction was applied, whereas to extract most hemicelluloses, the alkaline extraction method was used [4]. For subcritical water extraction, 2 g of 100–500 µm wood powder per line was extracted with 0.2 M formate buffer, pH 5.0, at 170 °C and 100 bar pressure in an accelerated solvent extractor (ASE-300, Dionex, USA). Extraction proceeded in three successive steps with residence times of 10, 20 and 30 min according to Sivan et al. [66]. Low-molecular weight compounds were removed by dialysis using Spectra/Por 3 membranes (Spectrum, USA), and the extracted polymers were freeze-dried. For the alkaline extraction, 1 g of wood powder with particle size 100–500 µm was incubated with 24% KOH for 24 h at room temperature [67, 68], filtered through 60 µm wire mesh and neutralized with 0.4 vol of acetic acid. Hemicelluloses were precipitated with 96% ethanol (4 °C for overnight), pelleted by centrifugation, washed in 80% ethanol, dissolved in distilled water and freeze-dried.

### Pyrolysis GC/MS

Approximately 50 µg (± 10 µg) of wood powder was used in a pyrolyzer (PY-2020iD and AS-1020E, Frontier Lab, Japan) connected to a GC/MS (7890A/5975C, Agilent Technologies Inc., Santa Clara, CA, USA) to analyze main wood components including carbohydrates, H-, G- and S-lignin units, and phenolic compounds, as described by Gerber et al. [69].

### Monosaccharide composition

For the wood samples, the 50–100 µm fraction of the powder was used in triplicates for monosaccharide analysis by a two-step sulfuric acid hydrolysis [70]. In brief, 1 mg of sample was incubated with 125 μL of 72% H_2_SO_4_ at room temperature for 3 h, then diluted with 1375 μL of deionized water and incubated at 100 °C for 3 h. Hydrolyzates were diluted 10 times with MilliQ water and filtered through a 0.2 mm syringe filter (Chromacol 17-SF-02-N) into HPAEC-PAD vials.

The sugar composition of sequential extracts containing non-cellulosic polysaccharides was determined following acidic methanolysis [71]. 1 mg of freeze-dried extract was incubated with 1 ml of 2 M HCl in dry methanol for 5 h at 100 °C. Samples were then neutralized with pyridine, dried under stream of air and further hydrolyzed with 2 M trifluoroacetic acid (TFA) at 120 °C for 1 h. The samples were then air-dried and dissolved in water. The sugar analysis was carried out by high-performance anion-exchange chromatography with pulsed amperometric detection (HPAEC-PAD) (ICS-6000 DC, Dionex) equipped with a CarboPac PA20 column (4 × 150 mm, Dionex) at 30 °C using the eluent gradients previously reported [66]. Samples were run in triplicates. Quantification of monosaccharides (monomeric form) was performed by standard calibration of ten monosaccharides (Ara, Rha, Fuc, Xyl, Man, Gal, Glc, GalA, mGlcA and GlcA) with concentrations between 0.005 and 0.1 g L^−1^.

### Acetyl content

The acetyl content of water extracts was determined in duplicates by overnight saponification of approx. 5 mg of sample in 1.2 mL of 0.8 M NaOH at 60 °C with constant mixing, neutralization with 90 µL of 37% HCl and filtration through 0.45 mm Chromacol syringe filters (17-SF-45(N), Thermo Fisher Scientific). The released acetic acid was detected by UV at 210 nm using high-pressure liquid chromatography with UV detector (Dionex-Thermofisher Ultimate 3100, USA) and separation by a Rezex ROA-organic acid column (300 × 7.8 mm, Phenomenex, USA) at 50 °C in 2.5 mM H_2_SO_4_ at 0.5 mL/min. Propionic acid was used as an internal standard.

### Oligosaccharide mass profiling (OLIMP)

For OLIMP analysis, the alkaline and 30 min water extracts were digested using GH30C *endo* β-(1–4) glucuronoxylanase (kindly provided by Prof. James F. Preston, University of Florida), and incubating 1 mg of extract in 1 mL of 20 mM sodium acetate buffer (pH 5.5) and 10 U enzyme for 16 h at 37 °C. After enzyme inactivation at 95 °C for 10 min, the hydrolysates were ten times diluted in acetonitrile 50% (v/v) with 0.1% (v/v) formic acid and filtered through Chromacol 0.2 μm filters (Scantec Nordic, Sweden). Samples were then briefly passed through a ZORBAX Eclipse Plus C18 column 1.8 μm (2.1 × 50 mm) (Agilent Technologies, Santa Clara, CA) and the oligosaccharide profiles were analyzed by a high-performance anion-exchange chromatography (HPAEC) system with pulsed amperometric detection (HPAEC-PAD) as reported previously [72] using xylo-oligosaccharides (X_2_-X_6_; Megazyme) as external standards and electrospray ionization mass spectrometry (ESI–MS) with a Synapt HDMS mass spectrometer (Waters, USA) in positive-ion mode and capillary and cone voltage set to 3 kV and 70 kV, respectively. The oligosaccharides were detected as [M + Na]+ adducts.

### Oligosaccharide separation and sequencing by tandem LC–MS/MS

Oligosaccharides obtained by GH30C hydrolysis of 30 min subcritical water extracts were derivatized by reductive amination with anthranilic acid, as previously described [73] and separated through an ACQUITY UPLC HSS T3 column (150 × 2.1 mm, Waters, USA) at a flow rate of 0.3 mL min^−1^ and a gradient of increasing acetonitrile content (10–30%) over 40 min. Mass spectrometric (MS) analysis was performed in positive mode with the capillary voltage and cone set to 3 kV and 70 kV, respectively. MS/MS was performed by selecting the m/z of interest through single ion monitoring (SIM) and subjecting it to collision-induced dissociation (CID) using argon as collision gas, at a ramped voltage of 35–85 V. Assignment of the proposed structures (Table S2) was performed by reference to labelled standards and analysis of the fragmentation spectra using ChemDraw (PerkinElmer, Waltham, Massachusetts, USA).

### Saccharification assay

Three technical replicates from each line and six from WT were used for analytical-scale saccharification. Wood powder moisture content was measured using Mettler Toledo HG63 moisture analyzer (Columbus, OH, USA) and 50 mg of dry material was used per sample. Acid pretreatment was carried out in an Initiator single-mode microwave instrument (Biotage Sweden AB, Uppsala, Sweden) using [1% (w/w) sulfuric acid] at 165 ˚C for 10 min and the enzymatic hydrolysis without or after acid pretreatment was performed at 45 °C using 4 mg of the liquid enzyme mixture Cellic CTec2 (cat. nr. SAE0020, Sigma-Aldrich, Saint Louis, MO, USA) as previously described [74]. Samples were analyzed for Glc production rate at 2 h using an Accu-Chek® Aviva glucometer (Roche Diagnostics Scandinavia AB, Solna, Sweden) following the calibration with a set of Glc standard solutions. After 72 h, the yields of monosaccharides were quantified using HPAEC-PAD (Ion Chromatography System ICS-5000 by Dionex, Sunnyvale, CA, USA) [75].

### Statistical analyses

Unless otherwise stated, statistical analyses (ANOVA followed by Dunnett’s test and contrast analysis) were performed in JMP Pro (v.16.0) software (SAS Institute Inc., Cary, NC, USA).

## Results

### Effects of transgenic modifications on plant growth and composition of wood cell wall and hemicelluloses

In our synthetic approach, we designed an RNAi vector construct targeting the two *Populus* homologs of *GUX1, PtGUX1A* and* B* [65], which are the two most highly expressed *GUX* genes during secondary wall biosynthesis in aspen [76] (https://plantgenie.org). The transgenic lines were denoted Gux1RNAi line 7 and 1ine 15A. For the post-synthetic reduction of glucuronidation, we used two transgenic lines expressing GH67 α-glucuronidase under control of wood-specific promoter (WP), denoted WP:GH67 line 8 and line 13 , which were described in the previous publication [50]. The transgenic lines did not exhibit any defect in growth and development under nine weeks of growth in the greenhouse (Figure S1a). Height and root growth of Gux1RNAi were similar to wild type (WT), whereas WP:GH67 lines grew better (Figure S1bc), as was previously reported [50]. RT-qPCR analysis of developing wood showed reduction in *PtGUX1A* expression in both analyzed lines, and *PtGUX1B* in line 7 (Figure S2).

**Fig. 1:**
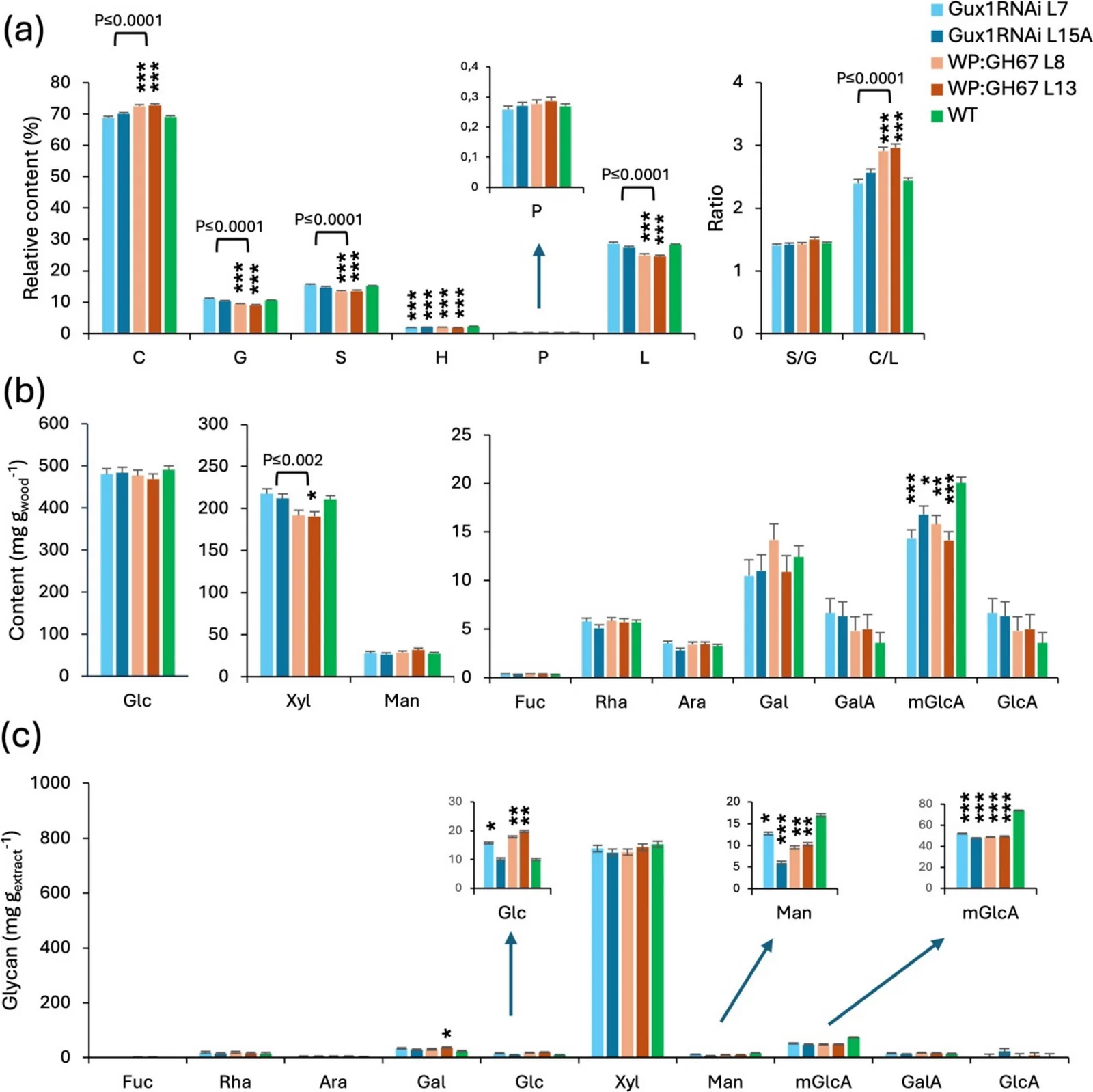
Effect of xylan modification during synthesis in the Golgi (Gux1RNAi) and post-synthetically *in muro* (WP:GH67) on chemical composition of wood. **a** Pyrolysis-GC/MS analysis showing relative content of carbohydrates (C), guaiacyl lignin (G), syringyl lignin (S), *p*-hydroxyphenyl lignin (H), phenolics (P), total lignin (L; G+S+H+P), and the derived ratios, S/G and C/L. **b** Wood monosaccharide composition determined by sulfuric acid hydrolysis. **c** Composition of alkali-extracted hemicelluloses. Data are means ± SE, *N* = 3 biological replicates for (a) and 3 technical replicates of pooled material from 6 trees for **b** and **c**. *P ≤ 0.05; **P ≤ 0.01; ***P ≤ 0.001 for comparisons with WT by post-ANOVA Dunnett’s test. P-values above the bars show significant difference between synthetic and post-synthetic modification (contrast analysis). Glucose (Glc), xylose (Xyl), mannose (Man), fucose (Fuc), rhamnose (Rha), arabinose (Ara), galactose (Gal), galacturonic acid (GalA), 4-*O*-methylglucuronic acid (mGlcA), glucuronic acid (GlcA)

The analysis of wood cell wall composition by Py-GC/MS showed changes in WP:GH67 lines, in which relative carbohydrate content increased, while the contents of guaiacyl (G), syringyl (S) and *p*-hydroxyphenyl (H) lignin (and the total lignin) decreased resulting in higher carbohydrate to lignin ratio (Fig. 1a). In contrast, cell wall composition in Gux1RNAi lines remained unchanged except a decrease in H-lignin units, which represent a minor lignin fraction only.

The wood monosaccharide content analyzed by sulfuric acid hydrolysis (Fig. 1b) did not show significant difference in monomers corresponding to major polysaccharides, such as glucose (Glc) or mannose (Man). A small decrease in Xyl was detected in WP:GH67 L13 line and consequently Xyl content was reduced in the WP:GH67 lines compared to the Gux1RNAi lines. The minor sugar fractions, such as fucose (Fuc), Rha, arabinose (Ara), galactose (Gal) and GalA, mostly reflecting the pectin content, were unaltered in both Gux1RNAi and WP:GH67 lines indicating that majority of sugar constituents of cell wall polysaccharides in the wood were affected by neither synthetic nor post-synthetic modification of xylan glucuronidation in aspen. In contrast, the content of mGlcA was significantly reduced in all transgenic lines confirming the reduction in xylan glucuronidation.

To further investigate changes in xylan glucuronidation level, we extracted hemicelluloses in alkaline conditions and analyzed their sugar composition. Their sugar composition confirmed the extraction of mainly glucuronoxylan (approx. 75% of Xyl and 5% of mGlcA with other sugars present in lower amounts; Fig. 1c) with preserved Xyl content in transgenic lines compared to WT and a decreased mGlcA content by 30–35%, confirming decrease in xylan glucuronidation. Changes in minor sugars, such as Man and Glc, were also recorded probably reflecting alteration in the extractability of hemicelluloses in transgenic lines because their overall sugar content of cell walls was not affected.

### Effects of synthetic and post-synthetic reduction of xylan glucuronidation on extractability of matrix polysaccharides by subcritical water

**Fig. 2:**
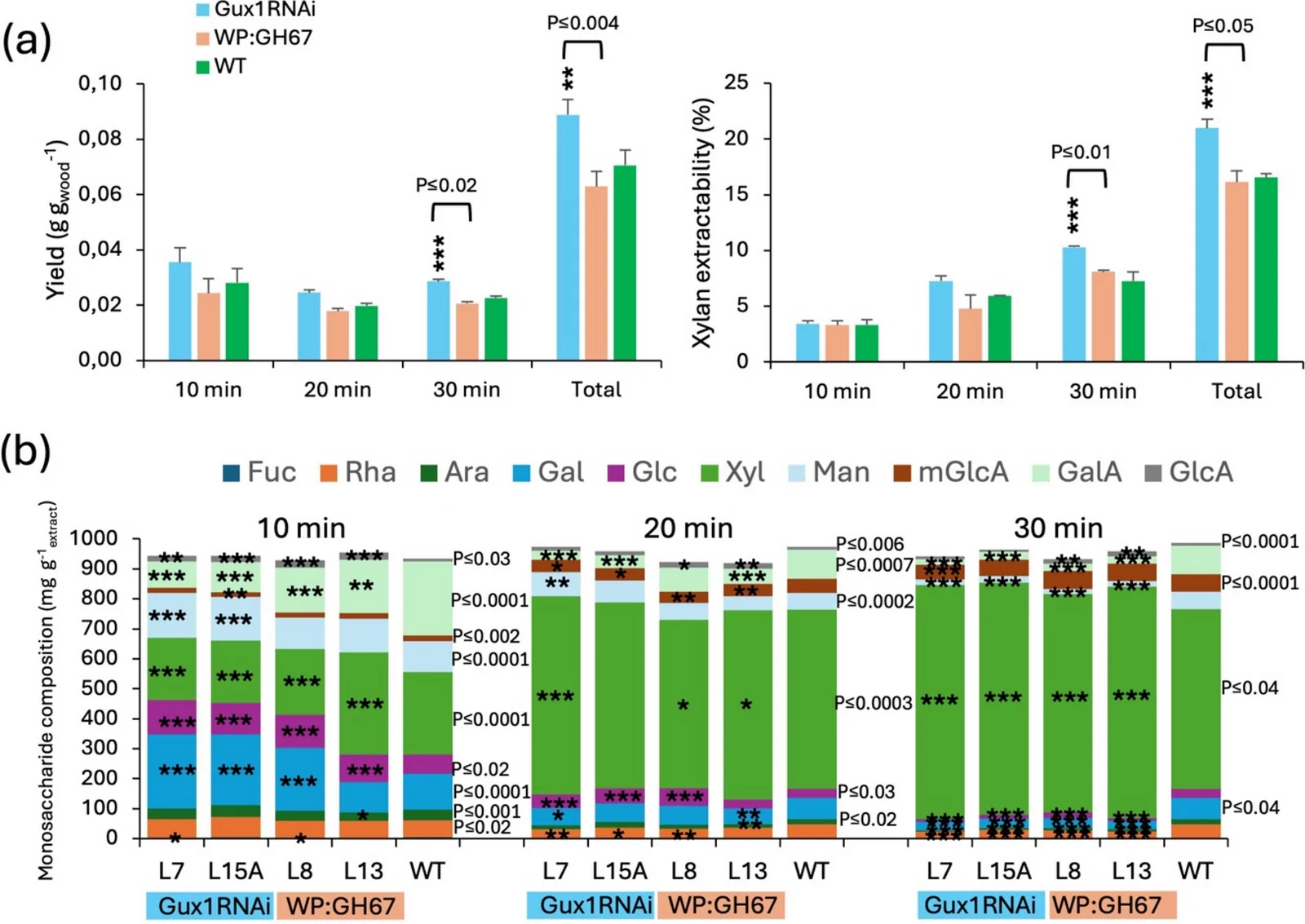
Subcritical water extraction and chemical characterization of cell wall polysaccharides in Gux1RNAi and WP:GH67 transgenic lines.** a** Yields of extract relative to wood weight and yields of xylan relative to starting xylan weight during successive steps of subcritical water extraction. **b** Monosaccharide composition of extracts. Data are means ± SE, *N* = 2 lines in **a**, and 2 technical replicates in **b**. *P ≤ 0.05; **P ≤ 0.01; ***P ≤ 0.001 for comparisons with WT by Dunnett’s test. *P*-values show significance of the differences between synthetic and post-synthetic modification (contrast analysis).

Subcritical water extraction (SWE) is meant to solubilize intact matrix polysaccharides from aspen wood, preserving their native acetylation [4, 66]. The total yield of the SWE process was between 6 and 9% of wood weight (Fig. 2a) and it was higher in Gux1RNAi than in WT or WP:GH67 lines. The main increase in the yield in Gux1RNAi lines was observed in 30 min extraction. The monosaccharide composition of sequential extracts determined by methanolysis-TFA analysis showed that 10 min extracts were enriched with pectic sugars (Gal, Ara, GalA and Rha), while longer duration extracts (i.e. 20 min and 30 min) were enriched in Xyl, thus assumed to contain mostly GX (Fig. 2b). However, distinct changes in the extract composition between WT and transgenic lines were noticed. In the 10 min extracts, all transgenic lines showed a higher content of Gal (except for WP:GH67 L13) and Glc, but a lower content of GalA, whereas Gux1RNAi lines had more Man compared to WT. In the 20 min extracts, relatively higher Man and Glc content was noticed in Gux1RNAi lines compared to WT and all transgenic lines had a reduced content of mGlcA. The 30 min extracts of transgenic lines showed significantly higher Xyl content, while the content of other sugar components, such as Gal, Ara, Rha, GalA, Glc and Man, were decreased compared to WT. The total xylan extractability determined as the content of Xyl in successive extracts relative to wood Xyl content revealed an approx. 30% increase in Gux1RNAi lines compared to WT, with approx. 40% increase in 30 min extracts, while no such trend was apparent in WP:GH67 lines (Fig. 2a).

We also noted many other differences in the composition of successive extracts between Gux1RNAi lines and WP:GH67 lines (shown by the P-values beside the bar graphs in Fig. 2b.). For example, the 10 min extracts differed in all sugars, but Fuc, the 20 min extracts differed in Xyl, Man and GalA and 30 min extracts in mGlcA and GlcA. These disparities indicated that extractabilities of GX and other cell wall polysaccharides were different between Gux1RNAi and WP:GH67 lines.

### Glucuronidation level and pattern in GUX1RNAi and WP:GH67 lines

**Fig. 3:**
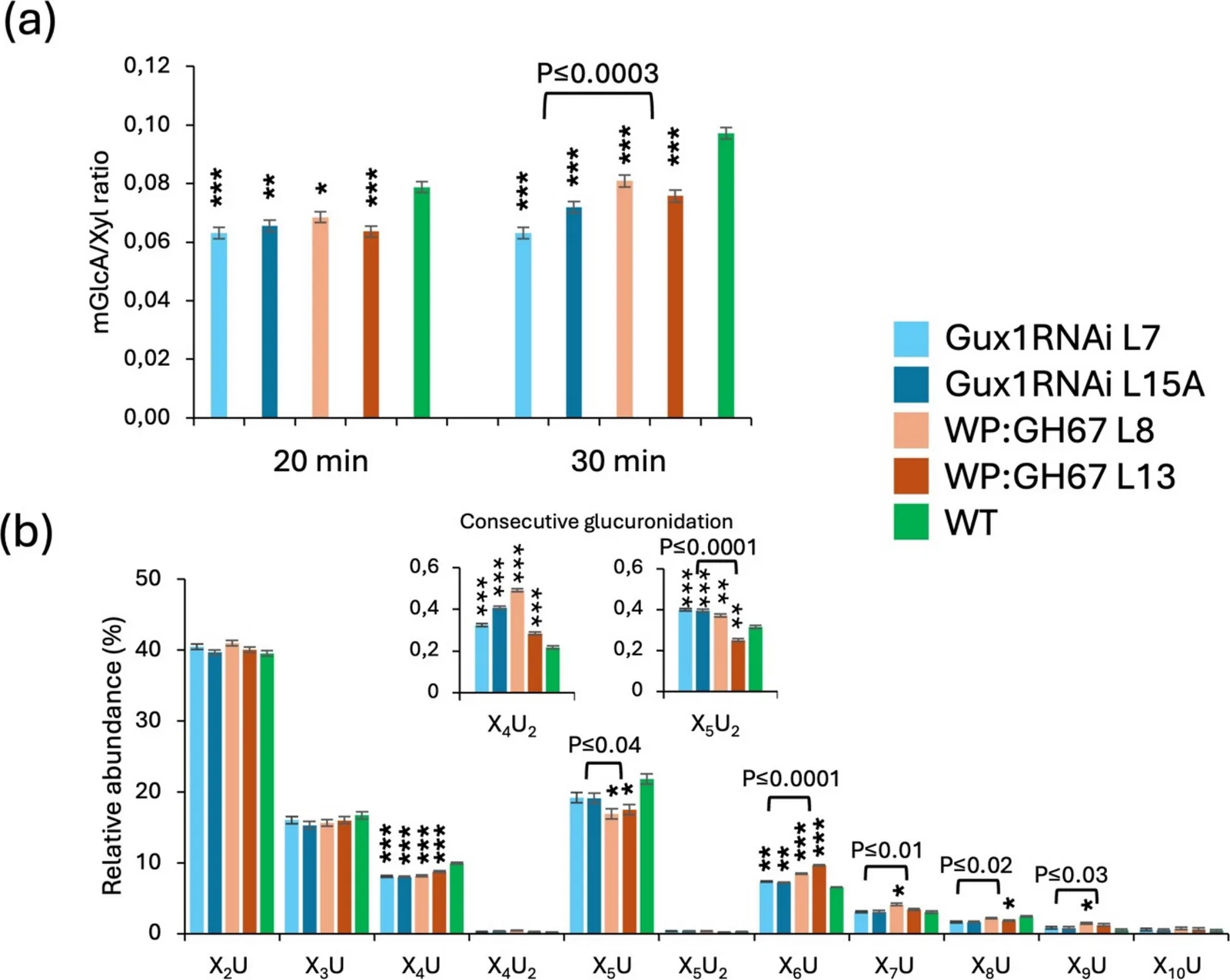
Xylan glucuronidation characteristics in the hemicellulose from Gux1RNAi and WP:GH67 transgenic lines.** a** Quantification of mGlcA/Xyl molar ratio in sequential 20 min and 30 min subcritical water extracts. **b** OLIMP profile showing relative abundance of uronidated xylo-oligosaccharides (UXO) released by GH30C glucuronoxylanase from the xylan extracted by alkaline method. Relative abundance calculated from the total ESI–MS intensities. Data are means ± SE, *N* = 3 in **a** and 2 in **b** technical replicates of wood powder pooled from 6 trees, *P ≤ 0.05; **P ≤ 0.01; ***P ≤ 0.001 for comparisons with WT by post-ANOVA Dunnett’s test. *P* values above the bars show significance of the differences between synthetic and post-synthetic modification (contrast analysis).

As the SWE preserves native xylan structure [4, 66] it is a method of choice for determination of GX glucuronidation level. The mGlcA/Xyl ratio in 20 min and 30 min subcritical extracts, containing primarily GX, was reduced in Gux1RNAi and WP:GH67 lines compared to WT (Fig. 3a), confirming the conclusions drawn from monosaccharide compositional analyses of wood and alkaline-extracted hemicelluloses (Fig. 1b, c), that both synthetic and post-synthetic GX modifications reduced glucuronic acid substitution of the xylan backbone. A subtle difference in glucuronidation level was observed in 30 min SWE extract between synthetic and post-synthetic modification. This fraction of GX, which represents a more tightly bound xylan than the 20 min fraction, was less glucuronidated in Gux1RNAi lines than in WP:GH67 lines (Fig. 3a).

To analyze the glucuronidation pattern, a more efficient alkali-extraction was used and xylo-oligosaccharides released by GH30C glucuronoxylanase from the extracts were analyzed by the high-performance anion-exchange chromatography with pulsed amperometric detection (HPAEC-PAD). All transgenic lines showed a decrease in peaks corresponding to uronidated xylo-oligosaccharides (UXOs) compared to WT (Figure S3). Mass spectrometry profiling of UXOs with specific spacing pattern showed that the xylan motifs representing even substitution, such as X_4_U, were reduced in Gux1RNAi lines, while WP:GH67 lines were affected in both odd and even substitution pattern represented by X_4_U and X_5_U oligosaccharides (Fig. 3b). Notably, the relative abundance of X_4_U_2_ motifs representing GX with consecutive glucuronidation and possibly interacting with lignin [3] were increased in all transgenic lines.

### Glucuronoxylan acetylation patterns in Gux1RNAi and WP:GH67 lines

**Fig. 4:**
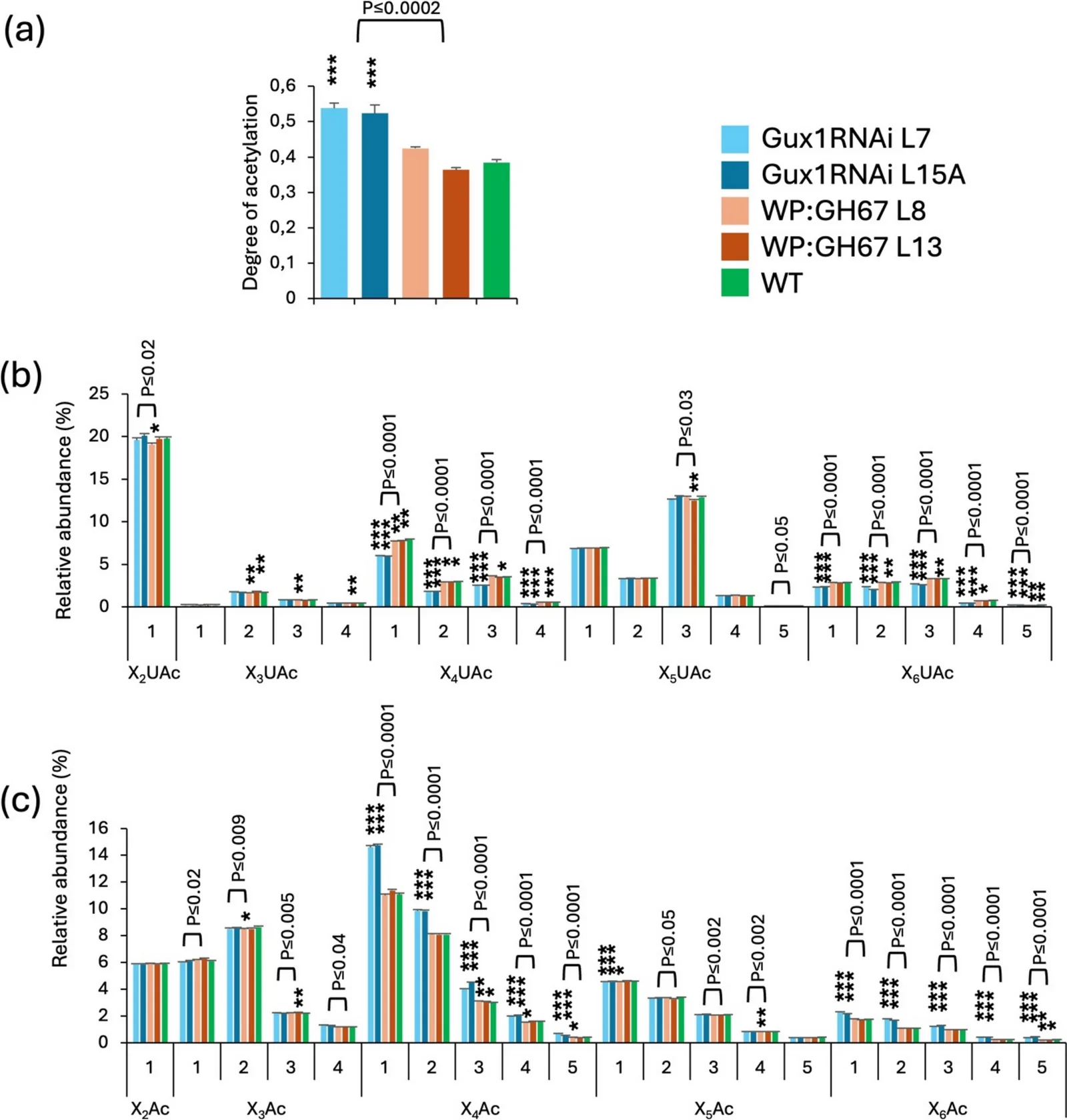
Characterization of xylan acetylation in 30 min SWE extracts of Gux1RNAi and WP:GH67 transgenic lines.** a** Degree of xylan acetylation shown as a molar ratio of acetyl to xylosyl units. **b** Relative abundance of uronidated xylo-oligosaccharides and **c** linear xylo-oligosaccharides released by GH30C glucuronoxylanase. Relative abundance calculated from the total ESI–MS intensities. The numbers below the X-axis show how many acetyl units are present in a given xylo-oligosaccharide. Data are means ± SE, *N* = 2 technical replicates, *P ≤ 0.05; **P ≤ 0.01; ***P ≤ 0.001 for comparisons with WT by Dunnett’s test. *P*-values above the bars show significance of the difference between synthetic and post-synthetic modification (contrast analysis).

GX acetylation was analyzed in 30 min SWE extracts because these extracts contained most highly purified GX. The degree of acetylation increased by approx. 40% in Gux1RNAi lines, while no change was observed in WP:GH67 (Fig. 4a). Profiling of uronidated xylo-oligomers released from the acetylated GX by GH30C glucuronoxylanase revealed a significant decrease in the relative content of acetylated oligomers corresponding to an even uronic acid substitution pattern, such as X_4_UAc_(n)_ and X_6_UAc_(n)_, with a concomitant increase of the corresponding non-uronidated xylo-oligosaccharides (X_4_Ac_(n)_, X_6_Ac_(n)_) in Gux1RNAi (Fig. 4b,c). This finding is in agreement with the *GUX1* genes being responsible for the major xylan domain with an even glucuronidation pattern. However, there was no consistent change in the abundance of the most prevalent X_2_UAc_1_ oligosaccharide that represents blocks of repeated X_2_UAc_1_ motif with acetylation and GlcA substitution present at *O*−3 and *O*−2 positions in the first Xyl, respectively [4]. This was consistent with unaltered abundance of X_2_U motif in alkali-extracted hemicelluloses (Fig. 3b). No major significant changes in acetylated oligomer motifs were found in WP:GH67 lines (Fig. 4b,c).

**Fig. 5:**
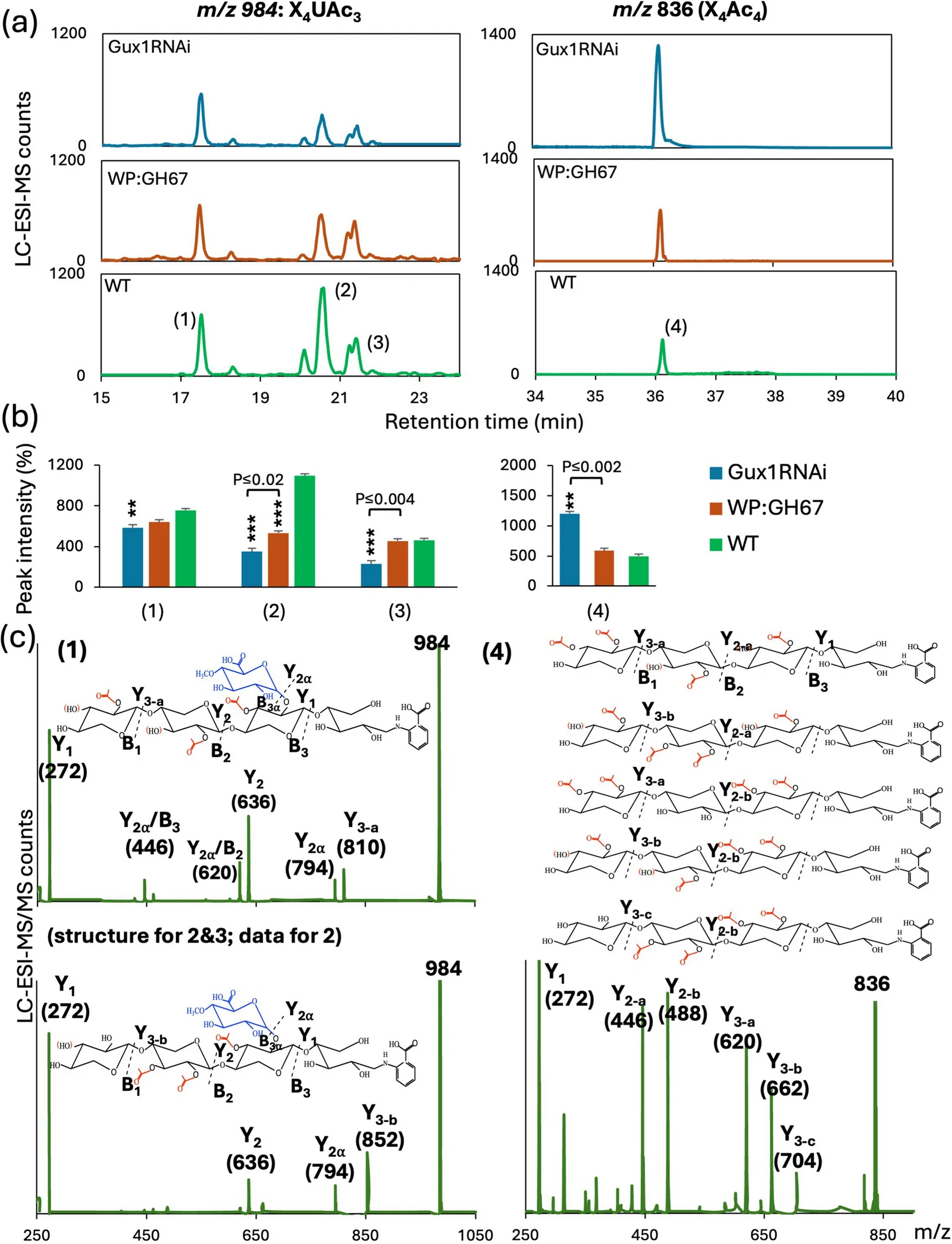
Oligosaccharide sequencing of glucuronoxylan showing dynamic changes in the *O*−2 glucuronidation and *O*−2, *O*−3 acetylation patterns in Gux1RNAi L15A and WP:GH67 L13 transgenic lines.** a** Single ion monitoring (SIM) chromatograms, **b** peak intensity of SIM and **c** fragmentation of X_4_UAc_3_ (m/z 984) and X_4_Ac_4_ (m/z 836) isomers by MS/MS and assignment of the ion fragments. X = Xylose; U = mGlcA; Ac = *O*-acetyl. Data in **b** are means ± SE, *N* = 2 technical replicates, *P ≤ 0.05; **P ≤ 0.01; ***P ≤ 0.001 for comparisons with WT by Dunnett’s test. P-values above the bars show significance of the difference between synthetic and post-synthetic modification (contrast analysis). Note that peak 1, 2 and 3 are isomers of X_4_UAc_3_ (m/z 984) while peak 4 corresponds to X_4_Ac_4_ (m/z 836)

To identify the position of acetylation that was increased in *GUX1*-suppressed plants, a glycan sequencing approach was used involving reducing end labeling of oligomers, single ion monitoring (SIM) for the separation of isobaric glucuronidated or acetylated xylo-oligomers followed by mass fragmentation. SIM analysis revealed several peaks corresponding to X_4_U, X_4_UAc_1_, X_4_UAc_2_ and X_4_UAc_3_. Analysis of X_4_UAc_1_, X_4_UAc_2_ (Figure S4) and X_4_UAc_3_ (Fig. 5) fragmentation showed that acetyl groups are always present on the *O*−3 position of Xyl units with mGlcA on *O*−2 position, confirming previous studies [3, 4]. Notably, a substantial decrease in the peak intensities corresponding to X_4_UAc_3_ (consecutive acetylation at *O*−2 in the two first xylosyl units and glucuronidation at *O*−2 with acetylation at *O*−3 in the third xylosyl unit; Fig. 5, data for peak 1) was noted in Gux1RNAi line 15A and in peak intensities corresponding to X_4_UAc_2_ (even acetyl spacing, Figure S4, peak 5) was apparent in both Gux1RNAi line 15A and WP:GH67 line 13. The SIM analysis of linear oligosaccharides X_4_Ac_1_, X_4_Ac_2_, X_4_Ac_3_ (Figure S5) and X_4_Ac_4_ (Fig. 5) revealed a substantial increase in the corresponding peak intensities, but only in Gux1RNAi line 15A (for example, for peaks 1, 2, 3, 7, 10, and 12; Figure S5). The peak intensity of single ion corresponding to X_4_Ac_4_ (representative oligomer with high acetylation; Fig. 5) and its mass fragmentation revealed that *O*−2, *O*−3 double acetylation is significantly increased in Gux1RNAi line 15A, but no change was evident in WP:GH67 line 13. The increase in *O*-*2* acetylation in X_4_Ac_n_ and in *O*−2, *O*−3 double acetylation in X_4_Ac_4_ in Gux1RNAi line 15A suggests that low GUX1 activity resulted in a possibility of higher *O*−2 occupancy by acetyl groups.

### Effects of synthetic and post-synthetic reduction in glucuronidation of xylan on biomass saccharification

**Fig. 6:**
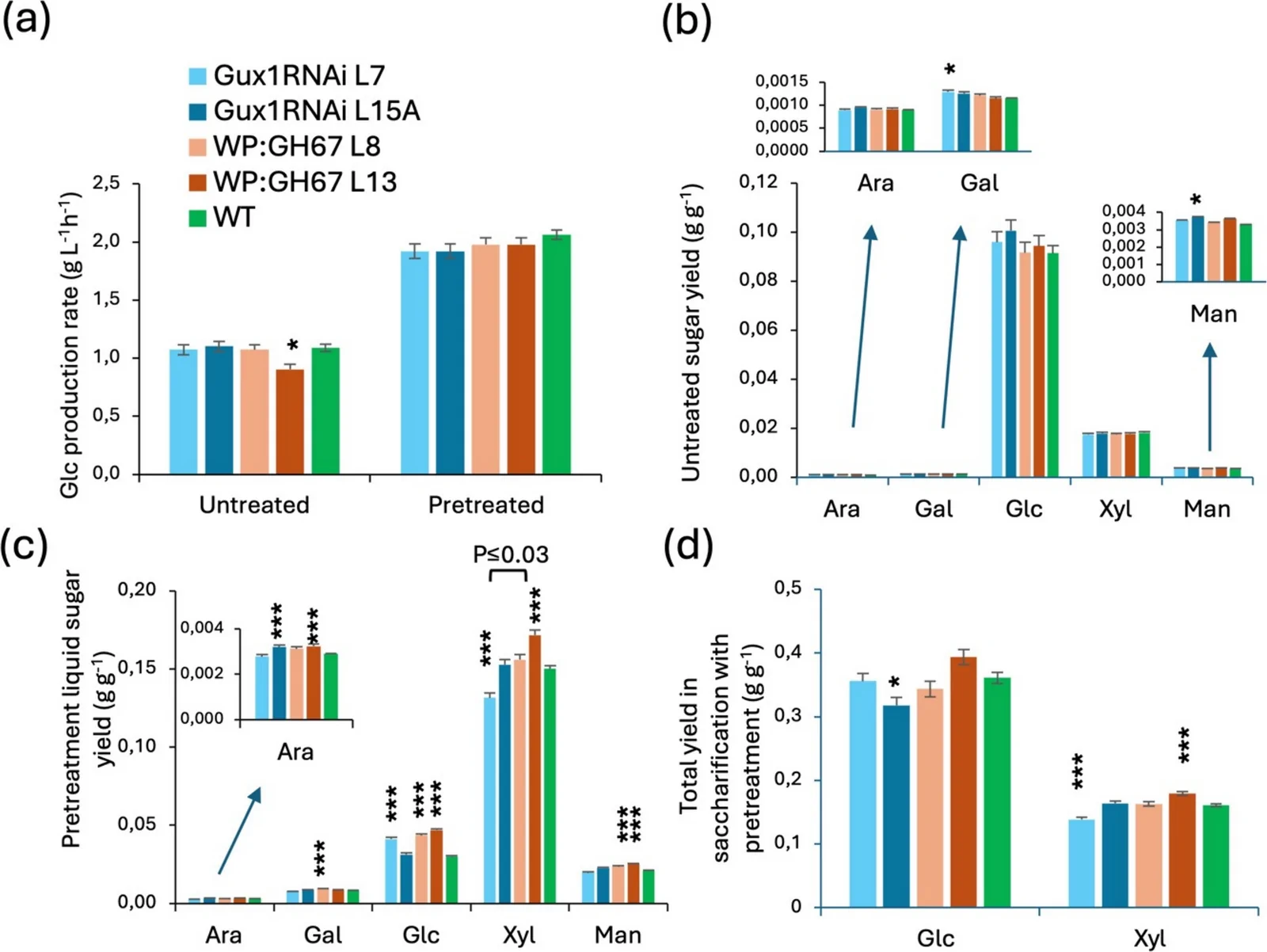
Saccharification of transgenic wood of Gux1RNAi and WP:GH67 lines. **a** Glucose production rates in saccharification without and with pretreatment. **b** Sugar yields in saccharification without pretreatment per g of wood. **c** Sugar yields in pretreatment liquid per g of wood. **d** Total glucose and xylose yields after acid pretreatment (yield of pretreatment liquid and enzymatic hydrolysis per g of wood). Data are means ± SE, N = 3 technical replicates of pooled wood samples from 6 trees. *P ≤ 0.05; **P ≤ 0.01; ***P ≤ 0.001 for comparisons with WT by post-ANOVA Dunnett’s test. P-value above the bars in **c** shows significance of the difference between synthetic and post-synthetic modification (contrast analysis). Glucose (Glc), xylose (Xyl), mannose (Man), fucose (Fuc), rhamnose (Rha), arabinose (Ara), galactose (Gal), galacturonic acid (GalA), 4-O-methylglucuronic acid (mGlcA), glucuronic acid (GlcA)

The saccharification of wood with and without acid pretreatment showed no consistent changes in the Glc production rates in either WP:GH67 or Gux1RNAi lines compared to WT (Fig. 6a). Sugar yields in saccharification without pretreatment were not consistently affected either (Fig. 6b). In the pretreatment liquid (diluted acid), an increase in the yield of Glc, Xyl and Man was noticed in the WP:GH67 lines and Glc in Gux1RNAi line 7 (Fig. 6c), indicating an increase in acid solubility of different matrix polysaccharides in these lines. In particular, higher Xyl yield was detected in WP:GH67 than in Gux1RNAi (P≤0.03) suggesting differences in xylan acid solubility between the synthetic and post-synthetic modifications. The total yields of Xyl and Glc in saccharification with pretreatment (pretreatment liquid yield and the following enzymatic hydrolysis yield) showed no consistent improvement in the transgenic lines (Fig. 6d). Our results indicate that the induced decrease in xylan glucuronic acid substitution did not alter the core molecular interactions within the cell wall that determine cellulose accessibility to hydrolytic enzymes during saccharification or that any hypothetical improvement is counteracted by some other indirect effects.

### Effects of synthetic and post-synthetic reduction in glucuronidation of xylan on transcriptomic changes in transgenic lines

**Fig. 7:**
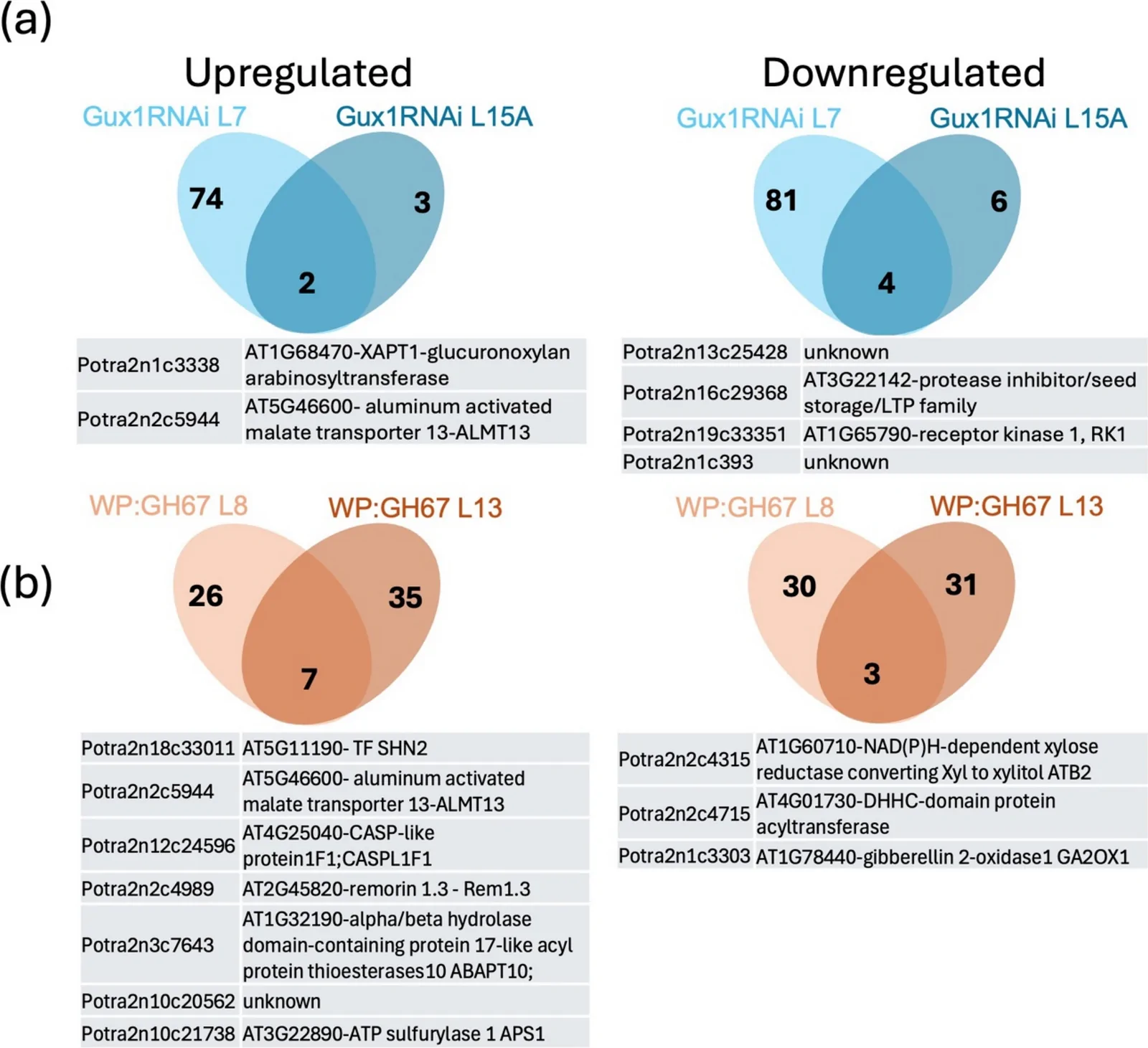
Number of differentially expressed (*P*_adj_ ≤0.05, |Log_2_(fold change)|≥ 0.58) genes shown as Venn diagrams in transgenic Gux1RNAi (**a**) and WP:GH67 lines (**b**), and lists of genes affected in common in both transgenic lines of each construct

To investigate if the decreased xylan glucuronidation could induce indirect effects involving changes in gene expression, RNA sequencing analysis of developing xylem was carried out in transgenic lines. The analysis revealed between 9 and 155 differentially expressed genes (DEGs) in Gux1RNAi lines of which only two and four were up- and downregulated, respectively, in common in both transgenic lines (Fig. 7a; Tables S3 and S4). Gene ontology (GO)/PFAM enrichment analysis of the commonly upregulated genes in Gux1RNAi lines revealed a significant upregulation in malate transfer and exostosin family (Table S6). These functions were represented by a homologue of *Arabidopsis* aluminium-activated malate transporter 13 (ALMT13), and the xylan biosynthetic gene XAPT1 encoding a glucuronoxylan arabinosyltransferase [28], respectively. No genes responsible for xylan acetylation were found upregulated, thus we did not find support for the hypothesis that an increase in acetylation seen in Gux1RNAi lines was due to an indirect effect involving an induction of acetylation-responsible genes.

In WP:GH67 lines, between 56 and 66 genes were differentially regulated with seven and three genes up- and downregulated, respectively, in common in both lines (Fig. 7b, Table S3 and S5). GO/PFAM enrichment analysis of upregulated genes indicated significant enrichment in domains of remorin, ATP-sulfurylase, AP2 and the malate transporter that was also found affected in the Gux1RNAi lines (Table S7). These functions were represented by single homologues of *Arabidopsis* genes *Rem1.3*, *APS1*, *ALMT13* and *SHN2* (Fig. 7b, Table S3), and indicated changes in membrane nanodomain organization, sulfate metabolism, malate transport and specific transcriptional regulation, respectively. The homologue of *CASPL1F1*, encoding a CASP-like protein and the homologue of *ABAPT10* encoding acyl protein thioesterase responsible for removing acylation were found upregulated, whereas the homologue encoding a DHHC-domain protein acyltransferase [77] was downregulated (Fig. 7b; Table S3). We also observed a downregulation of a homologue of *GA2OX1* encoding a GA19-inactivating oxygenase [78].

These results show that both synthetic and post-synthetic glucuronoxylan modification affected a malate transporter in common, but other changes in gene expression were different between Gux1RNAi and WP:GH67 lines.

## Discussion

### Synthetic and post-synthetic strategies of reducing xylan glucuronidation result in different mGlcA spacing patterns and cell wall chemistries

Both synthetic and post-synthetic strategies applied in this study were equally effective in reducing the level of glucuronidation by approx. 30–40%. However, the resulting pattern of glucuronidation was different between the two approaches: the suppression of *GUX1* genes resulted in changes in the major xylan domain as expected from the reported activities of GUX1 proteins [2, 6]; in contrast, expression of the GH67 α-glucuronidase affected both domains, especially the motifs, such as X_4_U and X_5_U, suggesting that GH67 α-glucuronidase might prefer these motifs due to their conformation.

The observed differences in glucuronidation patterns are expected to influence major interactions in cell wall [6, 11] and they could affect processes occurring post-synthetically *in muro,* such as lignin polymerization, potentially leading to different effects on cell wall composition (Fig. 1) and xylan extractability (Figs. 2, 6c).

However, the domain with mGlcA spacing on every other xylosyl unit represented by X_2_U and X_2_UAc motifs in the OLIMP analysis of deacetylated and acetylated GX (Figs. 3 and 4b), respectively, which forms arrays in aspen xylan [4, 66], was not affected by either approach except a slight decrease in line 8 of WP:GH67 construct. In Gux1RNAi lines, this could be explained by three hypotheses: (i) either the GUX1 enzymes are very effective in generating this motif, so that a reduction in primarily *GUX1A* expression achieved by RNAi suppression was not sufficient to affect this pattern or (ii) the pattern could be formed specifically by GUX1B (which transcripts were reduced merely by 46% and only in line 7) and not GUX1A protein or alternatively (iii) by another GUX protein not investigated here. In the WP:GH67 lines, negligent effect on the X_2_U motif indicates a hindrance specific to it. GH67 enzymes require the backbone hydrolysis before glucuronic acid, which is mediated by native plant GH10 enzymes [42, 43] exposing the side chain at the non-reducing ends. However, X_2_U arrays are inaccessible to GH10 hydrolases/transglycosylases that require at least two consecutive unsubstituted xylosyl units [79], and thus are protected from GH67 α-glucuronidases.

### Influence of synthetic and post-synthetic strategies of reducing xylan glucuronidation on xylan acetylation

The analysis of acetylated glucuronoxylan extracted by subcritical water showed that the suppression of *GUX1* genes, but not the expression of GH67 α-glucuronidase, led to increased xylan acetylation. OLIMP data also revealed that the xylan motifs with even substitution pattern were reduced for uronidated xylo-oligosaccharides, such as X_4_UAc_(n)_, X_6_UAc_(n)_ in Gux1RNAi lines, whereas the linear xylo-oligosaccharides corresponding to same spacing pattern, such as X_4_Ac_(n)_, X_6_Ac_(n)_ increased in abundance (Fig. 4). The fragmentation of the diagnostic xylo-oligosaccharides confirmed that the *O*−2,3 diacetylation increased when the synthesis of glucuronidation was suppressed (Fig. 5). Based on changes in the acetylation pattern observed in *gux1gux2* [57] and increased glucuronidation in the *esk1/tbl29* mutants [80], also much higher level of glucuronidation can be achieved by expressing the *GUX1* gene in the *esk1/tbl29* mutant than in the WT [81], it has been proposed that xylan acetyltransferase and glucuronyltransferase may compete with each other for the *O*−2 position of Xyl during biosynthesis of xylan in the Golgi. The increase in xylan acetylation observed in Gux1RNAi lines is in agreement with these previous proposals. Additionally, we demonstrated that there were no changes in expression of genes responsible for acetylation of xylan in transgenic lines, hence excluding any indirect effects and further supporting the proposed mechanism as the most likely cause of this phenotype.

In contrast to the suppression of *GUX1* genes, the expression of α-glucuronidase in secondary cell walls did not have any effect on the abundance of xylo-oligosaccharides with different acetylation patterns released by GH30C endoxylanase. Noteworthy, the expression of GH10 and GH11 endoxylanases *in planta*, which are partially restricted by acetylation and, therefore, preferably target low-acetylated domains, resulted in an increase in xylan acetylation [47].

### Glucuronidation changes did not improve lignocellulose biochemical conversion

Xylan glucuronidation is reported to play an important role in determining biomass recalcitrance due to its spacing pattern-based cellulose-xylan-lignin interaction [4, 8, 26]. The *gux1gux2* double mutation in *Arabidopsis* has been reported to enhance xylan alkali extractability and enzymatic conversion during saccharification [26, 54]. Moreover, the Glc yields of enzymatic saccharification were also increased, suggesting a potential for improving efficiency of the biochemical conversion of lignocellulosic biomass by reduction of xylan glucuronidation. Indeed, an approx. 50% reduction of xylan glucuronidation in sugar cane by suppression of *ScGUX2* gene resulted in consistent increase in Xyl yields and an increase in Glc yields in one of the three tested lines [55]. In the present study in aspen, we observed an increased xylan extractability by subcritical water in Gux1RNAi lines (Fig. 2), but not in WP:GH67 lines despite the similar level of glucuronidation decrease in both types of xylan modification. We argue that an increased acetylation observed in Gux1RNAi lines (but not in WP:GH67 line) (Fig. 4) could be responsible for better xylan solubility, as it is well known that there is an optimal xylan acetylation for maximum water solubility [82]. However, despite enhanced xylan extractability, no improvement in Glc production rate during saccharification with and without acid pretreatment was evident (Fig. 6). Indeed, the higher acetylation level of Gux1RNAi lines could act as a negative factor in saccharification [46, 53, 83, 84]. Similarly, no effect of reduced xylan glucuronidation on saccharification was observed when expressing α-glucuronidase and targeting it to cell walls (Fig. 6), confirming our results with 35S:GH67 lines [56]. These observations suggest that recalcitrance to cellulose accessibility by hydrolytic enzymes might be regulated in a more complex manner than by a simple decrease in glucuronidation. For instance, the blocks of X_2_U which is one of the main xylan domain motif in aspen tightly interacting with cellulose or domains with consecutive glucuronidation (motifs X_4_U_2_ and X_5_U_2_) interacting with lignin [4] were not reduced in the transgenic lines. It would be, therefore, interesting to study the role of these specific domains on biomass recalcitrance, but so far it is unknown which enzymes are responsible for their formation. Molecular simulations indicated that the degree, periodicity and position of acetylation at *O*−2 and *O*−3 as well as the distribution of glucuronidation can affect both the interaction of xylan with hydrophilic/hydrophobic cellulose surfaces and GX conformation [9, 10, 85, 86]. Consequently, the optimal structure of GX for the best cellulose accessibility still needs to be determined.

The reduced lignin content in WP:GH67 lines revealed by wood chemistry analysis (Fig. 1), had no positive effect on saccharfication yield indicating wood biomass recalcitrance is not only dependent on lignin content. It has been suggested that xylan covalent linkages to lignin could be responsible for recalcitrance. Indeed, the reduction in - γ ester links between glucuronic acid side chain of xylan and lignin by expressing fungal glucuronoyl esterase from family CE15 under control of WP improved saccharification, although it still could be debatable if this effect was directly linked to γ-ester linkage. reduction or to some indirect effects observed in the transgenic lines [48]. Expressing GH67 α-glucuronidase *in muro* does not affect γ-ester linkage, which could be explained by this linkage being formed inside the cells rather than in cell walls [56]. Consequently, saccharification was not improved by expressing GH67α-glucuronidase. On the other hand, repression of *GUX2* gene responsible for GlcA addition to the minor xylan domain, as in *gux1gux2 Arabidopsis* mutant [26, 54] or in transgenic sugarcane [55] might play a crucial role as this domain with a three-fold screw topology has been implicated in the interaction with lignin [87]. Thus, modification of genes responsible for specific interactions of xylan decorations with lignin might be necessary for improving the properties and biochemical conversion potential of hardwood trees.

### Synthetic and post-synthetic reductions in xylan glucuronidation lead to different changes in gene expression

Transcriptomics analysis provided some insights into two different patterns of plant response to modification of xylan glucuronidation during synthesis in the Golgi (suppression of *GUX1*) and in cell wall by targeting α-glucuronidase specifically to cells with SW deposition. Only one gene encoding a malate transporter was induced by both types of xylan modification (Fig. 7). Malate transport plays a crucial role in the regulation of turgor in plant cells [88, 89]. Such regulation might be critical in developing wood cells of transgenic lines since reduced glucuronidation is known to alter cell wall mechanical properties [26], which in turn would affect the turgor pressure.

The post-synthetic modification induced opposite changes in genes responsible for protein acylation, namely it induced *ABAPT10* responsible for de-acylation and suppressed DHHC-domain acyltransferase (Fig. 7). These changes indicate activation of processes aimed at decreasing protein S-acylation. Protein acylation is an important post-translational modification changing protein activity of diverse regulatory proteins including those related to receptors, signalling by calcium and G-proteins [77]. Therefore, the genes identified in WP:GH67 might play an important role in regulatory processes induced in transgenic lines. One such process is the secondary wall integrity sensing, which can induce growth in xylan-modified transgenic aspen under certain conditions [50]. The WP:GH67 L13 was one of the lines exhibiting increased growth in that study. Here, we show that another line of this construct, WP:GH67 L8 that also had slightly increased growth, showed induction of remorin *Rem1.3* and had a lower lignin content in cell walls similar to the other modified lines with increased growth [50]. Such phenotype could be mediated by SHN2, which was induced in both WP:GH67 lines (Fig. 7), because SHN2 is a key transcription factor inducing SW cellulose and xylan biosynthesis, while suppressing lignin biosynthesis in poplar [90]. Interestingly, a homologue of *CASPL1F1* encoding a CASP-like protein which restricts lignification to specific sites in cell wall during Casparian strip formation [91] was upregulated in WP:GH67 lines. These two upregulated genes could play a role in changes in cell wall composition in WP:GH67 lines, namely an increase in carbohydrates and decrease in lignin (Fig. 1). Other changes in gene expression in WP:GH67 lines could be related to activation of growth and stress responses, which are two physiological responses ultimately connected to secondary wall integrity signalling [50]. Specifically, a downregulation of *GA2OX1* would expectedly lead to increase in active gibberellins [92] and increased growth.

In conclusion, our study demonstrates that similarly decreased xylan glucuronidation by synthetic and post-synthetic strategies could lead to diverse responses, such as different patterns of xylan glucuronidation, acetylation, cell wall chemistry, gene expression and xylan extractability by subcritical water. The biosynthetic approach by suppression of *GUX1* genes led to an increase in xylan extractability and acetylation, decrease in even mGlcA substitution pattern, but no effect on expression of acetylation-related genes. The post-synthetic strategy by expressing GH67 α-glucuronidase in secondary cell walls caused a decrease in lignin content, in odd and even glucuronidation, and induced genes related to stress and growth. No improvement in Glc production rate during saccharification was detected in biomass from trees subjected to these modifications of xylan. Our study indicates that cellulose accessibility during biochemical conversion is influenced by more complex factors, such as the presence of specific xylan domains generating X_2_U(Ac), X_4_U_2_ and X_5_U_2_ oligosaccharides, which could maintain the cell wall architecture and its integrity by restricting access to cellulose for hydrolytic enzymes.

It is also apparent from the data obtained here that increase in the degree of xylan acetylation from approx. 0.3 to 0.5 does not appreciably affect saccharification, while improving xylan extractability by subcritical water by 20%. It would be possible then to design biomass with increased xylan acetylation where subcritical water extraction would be applied to pretreat biomass prior enzymatic saccharification [66].

## Supplementary Information

Additional file 1: Table S1. Primers used in the study. Table S2. Oligosaccharide assignment of the mass profiles obtained by ESI-MS. Table S3. Lists of all differentially expressed genes in each transgenic line. Table S4. Differentially expressed genes in wood-forming tissues of both Gux1RNAi transgenic lines compared to WT. Table S5. Differentially expressed genes in wood-forming tissues of both WP:GH67 transgenic lines compared to WT. Table S6. SignificantPFAM/GO enrichment for genes significantly upregulated or downregulated in common in both Gux1RNAi lines. Table S7. SignificantPFAM/GO enrichment for genes significantly upregulated or downregulated in common in both WP:GH67 lines

Additional file 2: Figure S1. Growth of Gux1RNAi and WP:GH67 transgenic lines. Figure S2. Relative expression of aspen *GUX1*, *GUX2* and *GUX3* genes in developing wood of transgenic Gux1RNAi lines. Figure S3. Analysis of xylan glucuronidation in Gux1RNAi and WP:GH67 lines

## Data Availability

The raw RNA-Seq data that support the findings of this study are available in the European Nucleotide Archive (ENA) at EMBL-EBI (https://www.ebi.ac.uk/ena/browser/home), under accession ID PRJEB82792.
