# Supplementary material for: Comparison of synthetic and post-synthetic molecular genetic methods of reducing xylan glucuronidation in hybrid aspen for biorefinery applications
Source: Biotechnol Biofuels Bioprod. 2026 Oct 15;19:82. doi: 10.1186/s13068-026-02830-x (PMC13670664; doi:10.1186/s13068-026-02830-x)
Supplement: Supplementary file 1 [file 13068_2026_2830_MOESM2_ESM.docx]

**Supplementary figures**

WT

WP:GH67

Gux1RNAi

(a)

(b)

Height (mm)

| Day after planting | WT | L7 | L15A | L8 | L13 |
| --- | --- | --- | --- | --- | --- |
| 15 | 106.25 | 108.333 | 117.5 | 107.5 | 113.333 |
| 17 | 124.583 | 126.667 | 135 | 128.333 | 129.167 |
| 19 | 149.583 | 155 | 156.667 | 151.667 | 150 |
| 22 | 191.875 | 206.667 | 215.833 | 209.167 | 213.333 |
| 24 | 236.25 | 241.667 | 252.5 | 252.5 | 265 |
| 26 | 271.042 | 285.833 | 293.333 | 287.5 | 299.167 |
| 28 | 311.458 | 326.667 | 332.5 | 337.5 | 350 |
| 30 | 350.208 | 364.167 | 367.5 | 380.833 | 385 |
| 32 | 393.333 | 402.5 | 410.833 | 428.333 | 446.667 |
| 34 | 447.083 | 450.833 | 465 | 476.667 | 504.167 |
| 36 | 499.375 | 510 | 514.167 | 532.5 | 561.667 |
| 38 | 550.417 | 558.333 | 564.167 | 589.167 | 610.833 |
| 40 | 600.417 | 607.5 | 609.167 | 651.667 | 670.833 |
| 42 | 653.542 | 658.333 | 667.5 | 708.333 | 741.667 |
| 44 | 715.625 | 722.5 | 710.833 | 762.5 | 800.833 |
| 46 | 767.917 | 772.5 | 780.833 | 830 | 859.167 |
| 48 | 823.333 | 829.167 | 839.167 | 891.667 | 914.167 |
| 50 | 883.958 | 894.167 | 901.667 | 960.833 | 978.333 |
| 52 | 942.083 | 945.833 | 959.167 | 1018.33 | 1061.67 |
| 54 | 1006.46 | 1012.5 | 1024.17 | 1092.5 | 1125.83 |
| 56 | 1071.67 | 1074.17 | 1090 | 1160 | 1192.5 |
| 58 | 1133.54 | 1138.33 | 1142.5 | 1228.33 | 1260 |
| 60 | 1198.75 | 1200.83 | 1201.67 | 1290 | 1330.83 |
| 63 | 1291.67 | 1290.83 | 1293.33 | 1384.17 | 1423.33 |

(c)

|  | Root dry weight (g) |
| --- | --- |
| Gux1RNAi L7 | 6.59333 |
| Gux1RNAi L15A | 8.515 |
| WP:GH67 L8 | 9.55 |
| WP:GH67 L13 | 9.18833 |
| WT | 7.02458 |

**Figure S1.** **Growth of Gux1RNAi and WP:GH67 transgenic lines**. **(a)** Representative images showing morphology of 9-weeks-old plants. Scale bar=10 cm. **(b)** Height growth and **(c)** root dry weight of 9-weeks-old plants. Data in (b) and (c) represent means for six transgenic trees per line and 24 wild type (WT) trees. Error bars in (c) represent SE and P-values show significant differences from WT based on the Dunnett’s test (not shown when ≥0.1).

Relative expression

|  | L7 | L15A | WT |
| --- | --- | --- | --- |
| GUX1A | 0.31157 | 0.30393 | 1.00448 |
| GUX1B | 0.54303 | 1.01497 | 1.01753 |
| GUX2 | 0.97903 | 1.08509 | 1.01792 |
| GUX3 | 1.15707 | 1.02832 | 1.01572 |

**Figure S2.** Relative expression of aspen *GUX1*, *GUX2* and *GUX3* genes in developing wood of transgenic Gux1RNAi lines. Expression was determined by RT-qPCR normalized to actin and ubiquitin genes. Means ± SE, n=3. P-values show significant differences between transgenic and WT samples (Dunnett’s test).

UXOs

(a)

Synthesis stage

PAD Signal (nC)

| Retention time (min) | WT | Gux1RNAi |
| --- | --- | --- |
| 0.000333 | 8.5953 | 8.6764 |
| 0.008667 | 8.606 | 8.53025 |
| 0.017 | 8.6021 | 8.80435 |
| 0.025333 | 8.56675 | 8.78595 |
| 0.033667 | 8.609 | 8.5363 |
| 0.042 | 8.60275 | 8.67995 |
| 0.050333 | 8.605 | 8.64525 |
| 0.058667 | 8.55845 | 8.7981 |
| 0.067 | 8.61815 | 8.4862 |
| 0.075333 | 8.60385 | 8.60425 |
| 0.083667 | 8.67345 | 8.64645 |
| 0.092 | 8.6642 | 8.5096 |
| 0.100333 | 8.5918 | 8.62705 |
| 0.108667 | 8.6201 | 8.62705 |
| 0.117 | 8.6084 | 8.40205 |
| 0.125333 | 8.63135 | 8.5681 |
| 0.133667 | 8.5907 | 8.6303 |
| 0.142 | 8.60815 | 8.614 |
| 0.150333 | 8.6016 | 8.5556 |
| 0.158667 | 8.5774 | 8.505 |
| 0.167 | 8.6276 | 8.483 |
| 0.175333 | 8.5645 | 8.52255 |
| 0.183667 | 8.65735 | 8.6052 |
| 0.192 | 8.5954 | 8.729 |
| 0.200333 | 8.60225 | 8.6507 |
| 0.208667 | 8.55405 | 8.50965 |
| 0.217 | 8.5571 | 8.48695 |
| 0.225333 | 8.57525 | 8.4802 |
| 0.233667 | 8.59425 | 8.51095 |
| 0.242 | 8.5426 | 8.6485 |
| 0.250333 | 8.692 | 8.77205 |
| 0.258667 | 8.5682 | 8.5681 |
| 0.267 | 8.56045 | 8.78855 |
| 0.275333 | 8.58835 | 8.6158 |
| 0.283667 | 8.58915 | 8.5685 |
| 0.292 | 8.5969 | 8.65435 |
| 0.300333 | 8.5771 | 8.73125 |
| 0.308667 | 8.61005 | 8.563 |
| 0.317 | 8.6269 | 8.4627 |
| 0.325333 | 8.5844 | 8.53645 |
| 0.333667 | 8.6175 | 8.6471 |
| 0.342 | 8.56555 | 8.5809 |
| 0.350333 | 8.58275 | 8.51055 |
| 0.358667 | 8.5858 | 8.5722 |
| 0.367 | 8.5756 | 8.3639 |
| 0.375333 | 8.57835 | 8.4615 |
| 0.383667 | 8.7354 | 8.57475 |
| 0.392 | 8.56435 | 8.749 |
| 0.400333 | 8.57495 | 8.31925 |
| 0.408667 | 8.5617 | 8.5495 |
| 0.417 | 8.5963 | 8.60255 |
| 0.425333 | 8.53135 | 8.5873 |
| 0.433667 | 8.6038 | 8.3452 |
| 0.442 | 8.6119 | 8.38125 |
| 0.450333 | 8.57705 | 8.29505 |
| 0.458667 | 8.614 | 8.59355 |
| 0.467 | 8.60805 | 8.38195 |
| 0.475333 | 8.65705 | 8.43465 |
| 0.483667 | 8.5848 | 8.50105 |
| 0.492 | 8.54605 | 8.39205 |
| 0.500333 | 8.61945 | 8.68605 |
| 0.508667 | 8.58065 | 8.5271 |
| 0.517 | 8.5023 | 8.3596 |
| 0.525333 | 8.5631 | 8.4425 |
| 0.533667 | 8.56535 | 8.47135 |
| 0.542 | 8.57865 | 8.37115 |
| 0.550333 | 8.55005 | 8.436 |
| 0.558667 | 8.60005 | 8.3284 |
| 0.567 | 8.56125 | 8.28675 |
| 0.575333 | 8.6 | 8.3942 |
| 0.583667 | 8.57835 | 8.93975 |
| 0.592 | 8.5857 | 8.5219 |
| 0.600333 | 8.60015 | 8.48505 |
| 0.608667 | 8.6179 | 8.38605 |
| 0.617 | 8.5935 | 8.38575 |
| 0.625333 | 8.551 | 8.31415 |
| 0.633667 | 8.624 | 8.5422 |
| 0.642 | 8.6091 | 8.59355 |
| 0.650333 | 8.67805 | 8.49 |
| 0.658667 | 8.5779 | 8.8524 |
| 0.667 | 8.56525 | 8.64405 |
| 0.675333 | 8.58575 | 8.76465 |
| 0.683667 | 8.5839 | 8.62595 |
| 0.692 | 8.64795 | 8.4059 |
| 0.700333 | 8.6198 | 8.50205 |
| 0.708667 | 8.57155 | 8.63615 |
| 0.717 | 8.62705 | 8.5605 |
| 0.725333 | 8.67245 | 8.32975 |
| 0.733667 | 8.57275 | 8.51525 |
| 0.742 | 8.56545 | 8.3786 |
| 0.750333 | 8.6024 | 8.45025 |
| 0.758667 | 8.65525 | 8.65195 |
| 0.767 | 8.56885 | 8.6744 |
| 0.775333 | 8.5998 | 8.4499 |
| 0.783667 | 8.5987 | 8.393 |
| 0.792 | 8.6168 | 8.45965 |
| 0.800333 | 8.59825 | 8.582 |
| 0.808667 | 8.61385 | 8.6218 |
| 0.817 | 8.62 | 8.43535 |
| 0.825333 | 8.56345 | 8.6331 |
| 0.833667 | 8.57195 | 8.5311 |
| 0.842 | 8.6162 | 8.6112 |
| 0.850333 | 8.68155 | 8.4271 |
| 0.858667 | 8.6059 | 8.5849 |
| 0.867 | 8.561 | 8.80585 |
| 0.875333 | 8.5576 | 8.284 |
| 0.883667 | 8.624 | 8.4592 |
| 0.892 | 8.56685 | 8.4177 |
| 0.900333 | 8.6205 | 8.4652 |
| 0.908667 | 8.5529 | 8.48745 |
| 0.917 | 8.62985 | 8.40875 |
| 0.925333 | 8.5663 | 8.76495 |
| 0.933667 | 8.5825 | 8.3849 |
| 0.942 | 8.6054 | 8.48635 |
| 0.950333 | 8.5868 | 8.5102 |
| 0.958667 | 8.6046 | 8.47035 |
| 0.967 | 8.63685 | 8.3847 |
| 0.975333 | 8.60435 | 8.4546 |
| 0.983667 | 8.6269 | 8.46725 |
| 0.992 | 8.57845 | 8.49075 |
| 1.00033 | 8.6175 | 8.54505 |
| 1.00867 | 8.60345 | 8.48095 |
| 1.017 | 8.6354 | 8.53555 |
| 1.02533 | 8.61305 | 8.6243 |
| 1.03367 | 8.56055 | 8.5904 |
| 1.042 | 8.60015 | 8.4848 |
| 1.05033 | 8.57665 | 8.42105 |
| 1.05867 | 8.62075 | 8.49715 |
| 1.067 | 8.5833 | 8.5298 |
| 1.07533 | 8.58025 | 8.5858 |
| 1.08367 | 8.55085 | 8.4921 |
| 1.092 | 8.55755 | 8.4541 |
| 1.10033 | 8.6256 | 8.6179 |
| 1.10867 | 8.57 | 8.53965 |
| 1.117 | 8.5844 | 8.49515 |
| 1.12533 | 8.61465 | 8.4372 |
| 1.13367 | 8.65835 | 8.5343 |
| 1.142 | 8.53865 | 8.5049 |
| 1.15033 | 8.591 | 8.6008 |
| 1.15867 | 8.58845 | 8.56165 |
| 1.167 | 8.5647 | 8.2798 |
| 1.17533 | 8.55365 | 8.50555 |
| 1.18367 | 8.6113 | 8.6489 |
| 1.192 | 8.59805 | 8.53 |
| 1.20033 | 8.5777 | 8.511 |
| 1.20867 | 8.6034 | 8.49315 |
| 1.217 | 8.627 | 8.58445 |
| 1.22533 | 8.59475 | 8.56915 |
| 1.23367 | 8.6067 | 8.5477 |
| 1.242 | 8.62705 | 8.80745 |
| 1.25033 | 8.585 | 8.8372 |
| 1.25867 | 8.64165 | 8.5334 |
| 1.267 | 8.58385 | 8.47115 |
| 1.27533 | 8.5591 | 8.4622 |
| 1.28367 | 8.6074 | 8.8015 |
| 1.292 | 8.58385 | 8.53385 |
| 1.30033 | 8.5748 | 8.41775 |
| 1.30867 | 8.6082 | 8.63685 |
| 1.317 | 8.58495 | 8.6729 |
| 1.32533 | 8.59305 | 8.56545 |
| 1.33367 | 8.65825 | 8.58895 |
| 1.342 | 8.5968 | 8.45055 |
| 1.35033 | 8.58365 | 8.51595 |
| 1.35867 | 8.6014 | 8.4806 |
| 1.367 | 8.6414 | 8.5219 |
| 1.37533 | 8.58025 | 8.8167 |
| 1.38367 | 8.6383 | 8.4103 |
| 1.392 | 8.6439 | 8.7022 |
| 1.40033 | 8.59025 | 8.407 |
| 1.40867 | 8.5914 | 8.5705 |
| 1.417 | 8.60635 | 8.46155 |
| 1.42533 | 8.572 | 8.47915 |
| 1.43367 | 8.6393 | 8.5766 |
| 1.442 | 8.58065 | 8.55405 |
| 1.45033 | 8.64275 | 8.5305 |
| 1.45867 | 8.569 | 8.91475 |
| 1.467 | 8.6187 | 8.4449 |
| 1.47533 | 8.59705 | 8.7112 |
| 1.48367 | 8.5873 | 8.38375 |
| 1.492 | 8.60205 | 8.44845 |
| 1.50033 | 8.59645 | 8.60095 |
| 1.50867 | 8.58735 | 8.5469 |
| 1.517 | 8.55725 | 8.72675 |
| 1.52533 | 8.6321 | 8.48515 |
| 1.53367 | 8.5415 | 8.5136 |
| 1.542 | 8.53915 | 8.40235 |
| 1.55033 | 8.4759 | 8.4069 |
| 1.55867 | 8.51375 | 8.435 |
| 1.567 | 8.53015 | 8.68395 |
| 1.57533 | 8.5688 | 8.71065 |
| 1.58367 | 8.5649 | 8.4836 |
| 1.592 | 8.5359 | 8.60565 |
| 1.60033 | 8.54345 | 8.5168 |
| 1.60867 | 8.57505 | 8.4504 |
| 1.617 | 8.54375 | 8.4908 |
| 1.62533 | 8.6234 | 8.3894 |
| 1.63367 | 8.63205 | 8.4133 |
| 1.642 | 8.61885 | 8.44405 |
| 1.65033 | 8.6895 | 8.473 |
| 1.65867 | 8.6963 | 8.49935 |

(b)

Post-synthesis stage

PAD Signal (nC)

| Retention time (min) | WT | WP:GH67 |
| --- | --- | --- |
| 0.000333 | 8.5953 | 8.44355 |
| 0.008667 | 8.606 | 8.51495 |
| 0.017 | 8.6021 | 8.49535 |
| 0.025333 | 8.56675 | 8.5017 |
| 0.033667 | 8.609 | 8.50095 |
| 0.042 | 8.60275 | 8.47615 |
| 0.050333 | 8.605 | 8.5278 |
| 0.058667 | 8.55845 | 8.5061 |
| 0.067 | 8.61815 | 8.51225 |
| 0.075333 | 8.60385 | 8.44445 |
| 0.083667 | 8.67345 | 8.5048 |
| 0.092 | 8.6642 | 8.49885 |
| 0.100333 | 8.5918 | 8.51945 |
| 0.108667 | 8.6201 | 8.50035 |
| 0.117 | 8.6084 | 8.4771 |
| 0.125333 | 8.63135 | 8.4639 |
| 0.133667 | 8.5907 | 8.41835 |
| 0.142 | 8.60815 | 8.51785 |
| 0.150333 | 8.6016 | 8.4695 |
| 0.158667 | 8.5774 | 8.47305 |
| 0.167 | 8.6276 | 8.53535 |
| 0.175333 | 8.5645 | 8.4594 |
| 0.183667 | 8.65735 | 8.5781 |
| 0.192 | 8.5954 | 8.5305 |
| 0.200333 | 8.60225 | 8.498 |
| 0.208667 | 8.55405 | 8.49335 |
| 0.217 | 8.5571 | 8.4831 |
| 0.225333 | 8.57525 | 8.50745 |
| 0.233667 | 8.59425 | 8.50745 |
| 0.242 | 8.5426 | 8.5127 |
| 0.250333 | 8.692 | 8.5023 |
| 0.258667 | 8.5682 | 8.49595 |
| 0.267 | 8.56045 | 8.52995 |
| 0.275333 | 8.58835 | 8.4868 |
| 0.283667 | 8.58915 | 8.47585 |
| 0.292 | 8.5969 | 8.46285 |
| 0.300333 | 8.5771 | 8.46635 |
| 0.308667 | 8.61005 | 8.46675 |
| 0.317 | 8.6269 | 8.41415 |
| 0.325333 | 8.5844 | 8.44975 |
| 0.333667 | 8.6175 | 8.4868 |
| 0.342 | 8.56555 | 8.42715 |
| 0.350333 | 8.58275 | 8.49165 |
| 0.358667 | 8.5858 | 8.48445 |
| 0.367 | 8.5756 | 8.4439 |
| 0.375333 | 8.57835 | 8.4566 |
| 0.383667 | 8.7354 | 8.47875 |
| 0.392 | 8.56435 | 8.52855 |
| 0.400333 | 8.57495 | 8.51555 |
| 0.408667 | 8.5617 | 8.43485 |
| 0.417 | 8.5963 | 8.45745 |
| 0.425333 | 8.53135 | 8.48225 |
| 0.433667 | 8.6038 | 8.42005 |
| 0.442 | 8.6119 | 8.49755 |
| 0.450333 | 8.57705 | 8.4255 |
| 0.458667 | 8.614 | 8.4906 |
| 0.467 | 8.60805 | 8.4839 |
| 0.475333 | 8.65705 | 8.5296 |
| 0.483667 | 8.5848 | 8.50265 |
| 0.492 | 8.54605 | 8.44235 |
| 0.500333 | 8.61945 | 8.4608 |
| 0.508667 | 8.58065 | 8.5118 |
| 0.517 | 8.5023 | 8.45205 |
| 0.525333 | 8.5631 | 8.47815 |
| 0.533667 | 8.56535 | 8.47075 |
| 0.542 | 8.57865 | 8.5069 |
| 0.550333 | 8.55005 | 8.5381 |
| 0.558667 | 8.60005 | 8.4476 |
| 0.567 | 8.56125 | 8.4583 |
| 0.575333 | 8.6 | 8.4406 |
| 0.583667 | 8.57835 | 8.48745 |
| 0.592 | 8.5857 | 8.50895 |
| 0.600333 | 8.60015 | 8.49985 |
| 0.608667 | 8.6179 | 8.4655 |
| 0.617 | 8.5935 | 8.50775 |
| 0.625333 | 8.551 | 8.4849 |
| 0.633667 | 8.624 | 8.5302 |
| 0.642 | 8.6091 | 8.49255 |
| 0.650333 | 8.67805 | 8.51135 |
| 0.658667 | 8.5779 | 8.463 |
| 0.667 | 8.56525 | 8.51915 |
| 0.675333 | 8.58575 | 8.47735 |
| 0.683667 | 8.5839 | 8.4945 |
| 0.692 | 8.64795 | 8.4907 |
| 0.700333 | 8.6198 | 8.47195 |
| 0.708667 | 8.57155 | 8.53755 |
| 0.717 | 8.62705 | 8.45885 |
| 0.725333 | 8.67245 | 8.4941 |
| 0.733667 | 8.57275 | 8.47375 |
| 0.742 | 8.56545 | 8.4659 |
| 0.750333 | 8.6024 | 8.4558 |
| 0.758667 | 8.65525 | 8.4802 |
| 0.767 | 8.56885 | 8.54915 |
| 0.775333 | 8.5998 | 8.44955 |
| 0.783667 | 8.5987 | 8.50985 |
| 0.792 | 8.6168 | 8.48185 |
| 0.800333 | 8.59825 | 8.50875 |
| 0.808667 | 8.61385 | 8.46605 |
| 0.817 | 8.62 | 8.55035 |
| 0.825333 | 8.56345 | 8.51335 |
| 0.833667 | 8.57195 | 8.54395 |
| 0.842 | 8.6162 | 8.5003 |
| 0.850333 | 8.68155 | 8.4957 |
| 0.858667 | 8.6059 | 8.4694 |
| 0.867 | 8.561 | 8.5662 |
| 0.875333 | 8.5576 | 8.5059 |
| 0.883667 | 8.624 | 8.52765 |
| 0.892 | 8.56685 | 8.5108 |
| 0.900333 | 8.6205 | 8.5156 |
| 0.908667 | 8.5529 | 8.4501 |
| 0.917 | 8.62985 | 8.568 |
| 0.925333 | 8.5663 | 8.4741 |
| 0.933667 | 8.5825 | 8.4483 |
| 0.942 | 8.6054 | 8.5372 |
| 0.950333 | 8.5868 | 8.549 |
| 0.958667 | 8.6046 | 8.50295 |
| 0.967 | 8.63685 | 8.46255 |
| 0.975333 | 8.60435 | 8.5473 |
| 0.983667 | 8.6269 | 8.55085 |
| 0.992 | 8.57845 | 8.5002 |
| 1.00033 | 8.6175 | 8.50435 |
| 1.00867 | 8.60345 | 8.5034 |
| 1.017 | 8.6354 | 8.553 |
| 1.02533 | 8.61305 | 8.4507 |
| 1.03367 | 8.56055 | 8.5045 |
| 1.042 | 8.60015 | 8.48445 |
| 1.05033 | 8.57665 | 8.5643 |
| 1.05867 | 8.62075 | 8.5247 |
| 1.067 | 8.5833 | 8.41015 |
| 1.07533 | 8.58025 | 8.5476 |
| 1.08367 | 8.55085 | 8.4726 |
| 1.092 | 8.55755 | 8.54765 |
| 1.10033 | 8.6256 | 8.5186 |
| 1.10867 | 8.57 | 8.5394 |
| 1.117 | 8.5844 | 8.5157 |
| 1.12533 | 8.61465 | 8.5447 |
| 1.13367 | 8.65835 | 8.53445 |
| 1.142 | 8.53865 | 8.52675 |
| 1.15033 | 8.591 | 8.52745 |
| 1.15867 | 8.58845 | 8.53905 |
| 1.167 | 8.5647 | 8.52865 |
| 1.17533 | 8.55365 | 8.52725 |
| 1.18367 | 8.6113 | 8.5375 |
| 1.192 | 8.59805 | 8.51775 |
| 1.20033 | 8.5777 | 8.46085 |
| 1.20867 | 8.6034 | 8.5621 |
| 1.217 | 8.627 | 8.5195 |
| 1.22533 | 8.59475 | 8.48295 |
| 1.23367 | 8.6067 | 8.5245 |
| 1.242 | 8.62705 | 8.5371 |
| 1.25033 | 8.585 | 8.53625 |
| 1.25867 | 8.64165 | 8.5249 |
| 1.267 | 8.58385 | 8.53405 |
| 1.27533 | 8.5591 | 8.4991 |
| 1.28367 | 8.6074 | 8.4448 |
| 1.292 | 8.58385 | 8.4889 |
| 1.30033 | 8.5748 | 8.547 |
| 1.30867 | 8.6082 | 8.4733 |
| 1.317 | 8.58495 | 8.5307 |
| 1.32533 | 8.59305 | 8.50285 |
| 1.33367 | 8.65825 | 8.48515 |
| 1.342 | 8.5968 | 8.4705 |
| 1.35033 | 8.58365 | 8.5139 |
| 1.35867 | 8.6014 | 8.55715 |
| 1.367 | 8.6414 | 8.5546 |
| 1.37533 | 8.58025 | 8.51695 |
| 1.38367 | 8.6383 | 8.52485 |
| 1.392 | 8.6439 | 8.45415 |
| 1.40033 | 8.59025 | 8.5661 |
| 1.40867 | 8.5914 | 8.51075 |
| 1.417 | 8.60635 | 8.51985 |
| 1.42533 | 8.572 | 8.5216 |
| 1.43367 | 8.6393 | 8.53725 |
| 1.442 | 8.58065 | 8.52725 |
| 1.45033 | 8.64275 | 8.55965 |
| 1.45867 | 8.569 | 8.5293 |
| 1.467 | 8.6187 | 8.5626 |
| 1.47533 | 8.59705 | 8.5602 |
| 1.48367 | 8.5873 | 8.53125 |
| 1.492 | 8.60205 | 8.5665 |
| 1.50033 | 8.59645 | 8.5729 |
| 1.50867 | 8.58735 | 8.48725 |
| 1.517 | 8.55725 | 8.5574 |
| 1.52533 | 8.6321 | 8.49335 |
| 1.53367 | 8.5415 | 8.40615 |
| 1.542 | 8.53915 | 8.4573 |
| 1.55033 | 8.4759 | 8.39555 |
| 1.55867 | 8.51375 | 8.4114 |
| 1.567 | 8.53015 | 8.37565 |
| 1.57533 | 8.5688 | 8.47575 |
| 1.58367 | 8.5649 | 8.51205 |
| 1.592 | 8.5359 | 8.515 |
| 1.60033 | 8.54345 | 8.4955 |
| 1.60867 | 8.57505 | 8.5181 |
| 1.617 | 8.54375 | 8.5263 |
| 1.62533 | 8.6234 | 8.4813 |
| 1.63367 | 8.63205 | 8.55875 |
| 1.642 | 8.61885 | 8.52355 |
| 1.65033 | 8.6895 | 8.5379 |
| 1.65867 | 8.6963 | 8.53945 |

UXO Area (nC min)

|  | UXO |
| --- | --- |
| Gux1RNAi | 1.3462 |
| WP:GH67 | 1.32655 |
| WT | 1.9025 |

**Figure S3.** **Analysis of xylan glucuronidation in Gux1RNAi and WP:GH67 lines.** **(a)** The oligosaccharide profiles from HPAEC-PAD analysis of xylo-oligosaccharides released by GH30C glucuronoxylanase from the alkali-extracted xylan. **(b)** Quantification of signals from uronidated xylo-oligomers (UXOs). Data are means ± SE, N = 2 technical replicates, *P ≤ 0.05; **P ≤ 0.01; ***P ≤ 0.001 for comparisons with WT by post-ANOVA Dunnett’s test.

**Y_3-b_**

**(768)**

(c)

(b)

X_4_UAc_2_ m/z 942

X_4_UAc_1_ m/z 900

X_4_U m/z 858

(a)

**Figure S4.** **Sequencing of uronidated xylan-oligosaccharides released from acetylated glucuronoxylan of Gux1RNAi L15A and WP:GH67 L13 transgenic lines by GH30C glucuronoxylanase.** **(a)** Single ion monitoring (SIM) chromatograms, **(b)** Peak intensity of SIM and **(c)** fragmentation of X_4_U (m/z 858, peaks 1-2), X_4_UAc (m/z 900, peak 3)_,_ X_4_UAc_2_(m/z 942, peaks 4-6) isomers by MS/MS including assignment of the ion fragments. X= Xylose; U= mGlcA; Ac= *O*-acetyl. Note the isomers of single m/z (appear as mutiple peaks) may corresponds to similar acetyl position, different acetyl position or mix. Data in (b) are means ± SE, N = 2 technical replicates, *P ≤ 0.05; **P ≤ 0.01; ***P ≤ 0.001 for comparisons with WT by Dunnett’s test. P-values above the bars show significance of the difference between synthetic and post-synthetic modification (contrast analysis).

X_4_Ac_3_ m/z 794

X_4_Ac_2_ m/z 752

X_4_Ac_1_ m/z 710

**Y_2-b_**

**(488)**

(b)

(c)

(a)

**Figure S5.** **Sequencing of linear xylan-oligosaccharides released from acetylated glucuronoxylan of Gux1RNAi L15A and WP:GH67 L13 transgenic lines by GH30C glucuronoxylanase.** **(a)** Single ion monitoring chromatograms, **(b)** Peak intensity of SIM and **(c)** fragmentation of X_4_Ac (m/z 710, peaks 1-4)_,_ X_4_Ac_2_(m/z 752, peaks 5-10)_,_ X_4_Ac_3_ (m/z 794, peaks 11-12) isomers by MS/MS including assignment of the ion fragments. X= Xylose; U= mGlcA; Ac= *O*-acetyl. Note the isomers of single m/z (appear as mutiple peaks) may corresponds to similar acetyl position, different acetyl position or mix. Data in (b) are means ± SE, N = 2 technical replicates, *P ≤ 0.05; **P ≤ 0.01; ***P ≤ 0.001 for comparisons with WT by Dunnett’s test. P-values above the bars show significance of the difference between synthetic and post-synthetic modification (contrast analysis).
